# Extensive genic and allelic heterogeneity underlying inherited retinal dystrophies in Mexican patients molecularly analyzed by next‐generation sequencing

**DOI:** 10.1002/mgg3.1044

**Published:** 2019-11-17

**Authors:** Juan C. Zenteno, Leopoldo A. García‐Montaño, Marisa Cruz‐Aguilar, Josué Ronquillo, Agustín Rodas‐Serrano, Luis Aguilar‐Castul, Rodrigo Matsui, Carlos I. Vencedor‐Meraz, Rocío Arce‐González, Federico Graue‐Wiechers, Mario Gutiérrez‐Paz, Tatiana Urrea‐Victoria, Ulises de Dios Cuadras, Oscar F. Chacón‐Camacho

**Affiliations:** ^1^ Department of Genetics Institute of Ophthalmology “Conde de Valenciana” Mexico City Mexico; ^2^ Department of Biochemistry Faculty of Medicine UNAM Mexico City Mexico; ^3^ AFINES Program Faculty of Medicine UNAM Mexico City Mexico; ^4^ Department of Retina Institute of Ophthalmology “Conde de Valenciana” Mexico City Mexico; ^5^ Vector Borne Disease Program Secretaría de Salud Pública Hermosillo Mexico

**Keywords:** Leber congenital amaurosis, next‐generation sequencing, retinal dystrophy, retinitis pigmentosa

## Abstract

**Background:**

Retinal dystrophies (RDs) are one of the most genetically heterogeneous monogenic disorders with ~270 associated loci identified by early 2019. The recent application of next‐generation sequencing (NGS) has greatly improved the molecular diagnosis of RD patients. Genetic characterization of RD cohorts from different ethnic groups is justified, as it would improve the knowledge of molecular basis of the disease. Here, we present the results of genetic analysis in a large cohort of 143 unrelated Mexican subjects with a variety of RDs.

**Methods:**

A targeted NGS approach covering 199 RD genes was employed for molecular screening of 143 unrelated patients. In addition to probands, 258 relatives were genotyped by Sanger sequencing for familial segregation of pathogenic variants.

**Results:**

A solving rate of 66% (95/143) was achieved, with evidence of extensive loci (44 genes) and allelic (110 pathogenic variants) heterogeneity. Forty‐eight percent of the identified pathogenic variants were novel while ABCA4, CRB1, USH2A, and RPE65 carried the greatest number of alterations. Novel deleterious variants in *IDH3B* and *ARL6* were identified, supporting their involvement in RD. Familial segregation of causal variants allowed the recognition of 124 autosomal or X‐linked carriers.

**Conclusion:**

Our results illustrate the utility of NGS for genetic diagnosis of RDs of different populations for a better knowledge of the mutational landscape associated with the disease.

## Graphical abstract

Extensive genic an allelic heterogeneity in retinal dystrophies in Mexican patients was identified. Genotyping of large cohorts of RD patiets from different ethnic groups is warranted for a better molecular knowledge of retinal degeneration.

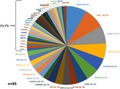

## 1 INTRODUCTION

The human retina is a specialized neural tissue in which the interaction of a variety of cell types allows the transduction of light stimuli into neural signals. The intrinsic molecular complexity of the retina directly correlates with the wide spectrum of acquired and genetic diseases that can affect retinal function. Retinal dystrophies (RDs) are an extensive group of inherited disorders arising from mutations in genes with a role in development, function, and maintenance of specific retinal cells (Tsui, Song, Lin, & Tsang, 2018). The collective frequency of RDs is about 1 in 2,000–2,500, with retinitis pigmentosa (RP) being the most common disease subtype (Broadgate, Yu, Downes, & Halford, 2017; Daiger, Sullivan, & Bowne, 2013; Sahel, Marazova, & Audo, 2014). RDs are the leading cause of genetic blindness among the working adult population and are the most common cause of inherited blindness worldwide (Francis, 2006). RDs can be classified in accordance with several criteria that include the primarily affected photoreceptor cell (cones, rods, or both), the age of onset of visual symptoms (congenital, juvenile, or adult), the evolution of disease (progressive or stationary), and the coexistence or not of extraocular anomalies (syndromic vs. non‐syndromic; see Berger, Kloeckener‐Gruissem, & Neidhart, 2010; Broadgate et al., 2017, for comprehensive reviews). While most RD cases initially exhibit localized dysfunction of a specific cell type, it is common that other retinal layers may be involved in advanced stages of disease, which can aggravate visual deficiency (Berger et al., 2010). In late stage disease, RD characterization based on funduscopic appearance is difficult because the entire retina might be compromised.

RDs are one of the most genetically heterogeneous human conditions as deleterious variants in approximately 270 autosomal or X‐linked genes have been demonstrated to be causal for these disorders (https://sph.uth.edu/retnet/, accessed on May 2019). RD gene products comprise proteins with roles not only in major retinal processes as phototransduction, retinoid recycling pathway, function and maintenance of the connecting cilium, or retinal development (Inglehearn, 1998), but also proteins with dissimilar functions as RNA splicing (Růžičková & Staněk, 2017), extracellular matrix integrity (Eudy et al., 1998), and lipid metabolism (Agbaga et al., 2008).

The identification of the mutated gene is critical for improving the care of RD patients and their families by allowing a precise disease classification and providing valuable prognostic information. In addition, recognition of the defective gene in young patients allows for a better planning of medical care by anticipating extraocular complications that occur in a number of syndromic RDs (Chacón‐Camacho, Garcia‐Montaño, & Zenteno, 2017; Sadagopan, 2017).

In recent years, the application of next‐generation sequencing (NGS) techniques has greatly improved the molecular characterization of RDs. NGS allows the simultaneous screening of the entire set of known genes causing RDs (gene panels), the systematic analysis of the coding regions of all genes (whole exome sequencing), or even the sequencing of the entire content of a patient's genome (whole genome sequencing). As NGS costs continue to decrease, it is anticipated that its application in clinical settings will be substantially expanded. NGS has been recently applied for the recognition of pathogenic variants in cohorts of RDs patients from different ethnicities, with rates of molecularly solved cases ranging from 50% to 75% (Bernardis et al., 2016; Birtel et al., 2018; Boulanger‐Scemama et al., 2015; Bravo‐Gil et al., 2016; Di Iorio et al., 2017; Ge et al., 2015; Patel et al., 2016; Riera et al., 2017; Wang et al., 2014; Zhao et al., 2015). Differences in rates of NGS‐solved cases are presumably due to several aspects such as the inclusion of sporadic versus familial cases, the type of NGS approach (gene panels vs. exome or genome sequencing), and features inherent to the particular mutational profile of a RD population (frequency of consanguineous marriages or occurrence of founder mutation effects). Molecular characterization of additional RD cohorts is warranted as they will allow the expansion of the spectrum of pathogenic variants linked to human retinal degeneration, will permit the identification of additional genotype–phenotype correlations, and will recognize ethnic‐specific founder effects which would facilitate subsequent molecular analysis in patients from such populations.

In this work, we applied NGS to an extensive group of RD patients from Mexico, a previously uncharacterized population. A diagnostic rate of 66% was obtained (95/143 probands) and a total of 110 distinct pathogenic variants (53 of them novel) in 44 known RD genes were identified.

## 2 MATERIALS AND METHODS

### 2.1 Recruitment and clinical data of RD patients

The study was approved by the institutional review board of the “Conde de Valenciana” Institute of Ophthalmology (Mexico City, Mexico) and adhered to the tenets of the Declaration of Helsinki. Written informed consent was obtained from all the participants or their parents when indicated. A total of 143 unrelated probands with different forms of RD were enrolled in the study. All probands underwent a complete ophthalmological evaluation, including best corrected visual acuity, biomicroscopy, color fundus photography, fundus autofluorescence, electrophysiological testing, and swept‐source optical coherence tomography (DRI SS OCT Triton, (TOPCON corporation)). The clinical diagnosis of a RD was made by retina specialists based on the patient's history of visual symptoms, clinical examination, retinal imaging, and electrophysiology. Clinical and family history details were collected during genetic consultation. Genetic analysis procedures were performed at the Laboratory of Molecular Genetics of the same Institute in Mexico City. Blood samples from available affected and unaffected family members were collected for co‐segregation analysis. The mode of inheritance was assumed to be autosomal recessive in case of parental consanguinity or if only siblings were affected, autosomal dominant if the family history was suggestive for the same retinal disease in at least two successive generations with males and females similarly affected, and X‐linked if the disease occurred in different generations without male‐to‐male transmission and with males being severely affected while females were normal or with only minor symptoms. If other family members were affected but the pedigree was not suggestive for any of the above patterns, inheritance was classified as inconclusive. Sporadic or simplex occurrence of the RD was assumed in families with a single affected individual and no history of parental consanguinity. Systemic examinations were performed by a geneticist in order to identify syndromic RD cases.

### 2.2 Targeted sequencing panel

Gene enrichment during library preparation was performed using the commercial ClearSeq Inherited Disease Panel (Agilent Technologies). This panel has a 10.5 Mb capture capacity and targets the coding exons and splicing regions of 2,742 genes involved in inherited disorders. The panel includes 199 RD‐related genes, in accordance with the Retinal Information Network (RETNET; https://sph.uth.edu/retnet/home.htm) and the Online Mendelian Inheritance in Man (OMIM) catalog (https://www.omim.org/ ) at the time of the study (accessed on September 2018). One hundred and four of these genes have been implicated exclusively in non‐syndromic RDs, 23 are associated with both syndromic and non‐syndromic forms of RDs, and 72 are involved only in syndromic RDs (Table S1).

### 2.3 DNA extraction, library preparation, and sequencing

Genomic DNA (gDNA) was extracted from peripheral blood leukocytes of all participants using the QIAaMP DNA Blood kit (Qiagen), following the manufacturer's protocol. gDNA integrity was assessed through gel electrophoresis and initial quantification and purity of samples were measured employing a Nanodrop 2000 spectrophotometer (Thermo Fisher Scientific). Libraries were constructed using the SureSelect QXT system (Agilent Technologies) and enrichment was performed using the ClearSeq Inherited Disease hybridization capture panel (Agilent Technologies). Briefly, gDNA was quantified and adjusted to a 24 ng/ul dilution, using the Qubit dsDNA High Sensitivity kit (Invitrogen) and a Qubit 2.0 fluorometer (Invitrogen). Subsequently, 50 ng of gDNA was enzymatically fragmented and DNA fragments were purified, amplified, and hybridized to the capture panel. For each sample, index adaptors were ligated to the 5´ and 3´ ends. DNA fragments were re‐amplified by PCR, and the fragments from 300 to 500 bp were isolated. All fragment purifications were performed using the Agencourt AMPure XP kit (Beckman Coulter Genomics), and the quality of the libraries was assessed through a Bioanalyzer 2100 (Agilent Technologies) using the High Sensitivity DNA kit (Agilent Technologies). Lastly, libraries were pooled in groups of five and sequenced in a MiSeq NGS platform (Illumina) at a concentration of 10 p.m. using the MiSeq Reagent kit v2 300 cycles (Illumina).

### 2.4 NGS Data Processing and variant identification

Data obtained from sequencing runs in the form of FASTQ files were processed using the EMC Galaxy Server. Briefly, FASTQ files were converted to a FASTQ Sanger format using the tool FASTQ Groomer. Sequence data were read and mapped using the Burrows‐Wheeler Aligner (BWA)‐MEM algorithm. VarScan 2.3.6 was employed for variant calling, generating two variant call format (VCF) files per sample, one for single nucleotide variants (SNVs) and another one for insertions and deletions (INDELS). The GRCh37 (hg19) was used as the reference genome sequence for mapping and variant calling (https://bioinf-galaxian.erasmusmc.nl/galaxy/). For average coverage and read depth analysis, gene coordinates of targeted regions were obtained from UCSC Genome Browser, according to the hg19 reference genome. Aligned read calculations were performed using SAMStat (http://samstat.sourceforge.net/), and coverage of the targeted regions was calculated through Bedtools (https://github.com/arq5x/bedtools2/).

For causal variants detection, each VCF file was filtered and reviewed using the VariantStudio 3.0 software (Illumina). Variants were annotated for minor allele frequencies (MAFs) in the 1,000 genomes (Abecasis et al., 2012), Exome Variant Server (NHLBI GO Exome Sequencing Project), and Exome Aggregation Consortium (ExAC) databases. Variants with MAF > 0.05 and read depth < 10× were filtered out. Pathogenicity of candidate variants was assessed based on a number of criteria, which included reports on published literature and severe effects on protein function (stop‐gained, stop‐lost, frameshift, and start‐lost variants). For missense variants, in silico prediction programs as Polymorphism Phenotyping v2 (PolyPhen‐2; Adzhubei et al., 2010), Sorting Intolerant From Tolerant (SIFT; Kumar, Henikoff, & NG, 2009), and Mutation Taster 2 (Schwarz, Cooper, Schuelke, & Seelow, 2014) were employed for pathogenicity prediction. Additionally, five different algorithms were used by Alamut Visual 2.10‐0 (Interactive Biosoftware, Rouen, France, http:\\www.interactive-biosoftware.com) to predict the splicing effect of variants located in the flanking regions of exons and for deep‐intronic variants. For copy number variant (CNV) detection, coverage results from all RD genes were exported to an excel document and screened for regions with no coverage. When regions with zero reads were identified in a sample, further analysis was performed by visualizing BAM files on the Integrative Genomic Viewer (IGV, Broad Institute; http://software.broadinstitute.org/software/igv/). These regions were compared across different samples from the same sequencing run to discard problems in coverage due to technical factors. This approach allowed us to detect large homozygous or hemizygous intragenic deletions, but was not efficient for heterozygous CNV recognition. Initial variant screening was directed to the 199 RD‐related genes included in the sequencing panel (Table S1). However, in unsolved cases, the analysis was extended to non‐RD genes, employing the above mentioned criteria for pathogenicity assessment of variants. Novel candidate variants identified as pathogenic were screened in a set of 100 in‐house clinical exomes.

### 2.5 Confirmation and segregation analysis of variants by Sanger sequencing

All candidate pathogenic variants were confirmed by Sanger sequencing and co‐segregation analysis was subsequently performed on available family members to further support their pathogenicity. For Sanger sequencing, primers for polymerase chain reaction (PCR) were designed using the Primer‐BLAST program from the National Center for Biotechnology Information (NCBI) and are available upon request. PCR products were purified and sequenced using the BigDye Terminator Cycle sequencing kit (Applied Biosystems Foster City, CA). All samples were analyzed using a 3130 Genetic Analyzer (Applied Biosystems).

### 2.6 RNA extraction and cDNA synthesis for *BBS9* transcriptional analysis

Total RNA was extracted from saliva following the method described by Pandit, Cooper‐White, and Punyadeera (2013). Cell pellets from 1 ml of saliva samples were used for RNA extraction. RNA samples were treated with RNAse‐free DNase I (QIAGEN) in accordance with the manufacturer's protocol. Reverse transcription was performed using the Superscript III First Strand Synthesis System (Thermo‐fisher). PCR was carried out with 300 ng of cDNA, using the primers that spanned candidate splicing regions. Primers are available upon request. Amplicons were gel purified and sequenced using the BigDye Terminator Cycle Sequencing Kit and a 3130 Genetic Analyzer (Applied Biosystems).

## 3 RESULTS

### 3.1 Patients

A total of 143 probands with different forms of RDs, including 124 (87%) non‐syndromic and 19 (13%) syndromic cases were ascertained (Table 1). According to the pedigree structure and clinical data, autosomal recessive retinitis pigmentosa (ARRP) was assumed in 37 probands, autosomal dominant retinitis pigmentosa (ADRP) in 16, X‐linked RP (XLRP) in 3, and sporadic or simplex RP in 29. In addition, Leber congenital amaurosis (LCA) was clinically diagnosed in 21 probands, cone‐rod dystrophy (CRD) in 7, and macular dystrophy (MD)/Stargardt disease (STGD) in 11 (Table 1). Syndromic ARRD was diagnosed in 12 unrelated patients while simplex syndromic RD was established in 7. All patients included in this study were of Mexican‐mestizo descent and originated mainly from central and southern Mexico.

**Table 1:** Clinical diagnosis in 143 unselected cases of retinal dystrophies

Type of RD	# of cases	Percentage
ARRP	37	26
ADRP	16	11
Simplex RP	29	20
LCA	21	15
CRD	7	5
MD/STGD	11	8
Syndromic ARRD	12	8
Simplex syndromic RD	7	5
X‐linked RP	3	2
Total	143	100

Abbreviations: ADRP, Autosomal Dominant Retinitis Pigmentosa; ARRD, Autosomal Recessive Retinal Dystrophy; ARRP, Autosomal Recessive Retinitis Pigmentosa; CRD, Cone‐Rod Dystrophy; LCA, Leber Congenital Amaurosis; MD, Macular Disease; RD, Retinal Dystrophy; RP, Retinitis Pigmentosa; STGD, Stargardt Disease.

### 3.2 Targeted NGS coverage

Coverage and read depth analysis indicated that, on average, 99.45% of the targeted regions for the analyzed RD genes were covered with an average read depth of 92X.

### 3.3 Identification of pathogenic variants

**Figure 1:**
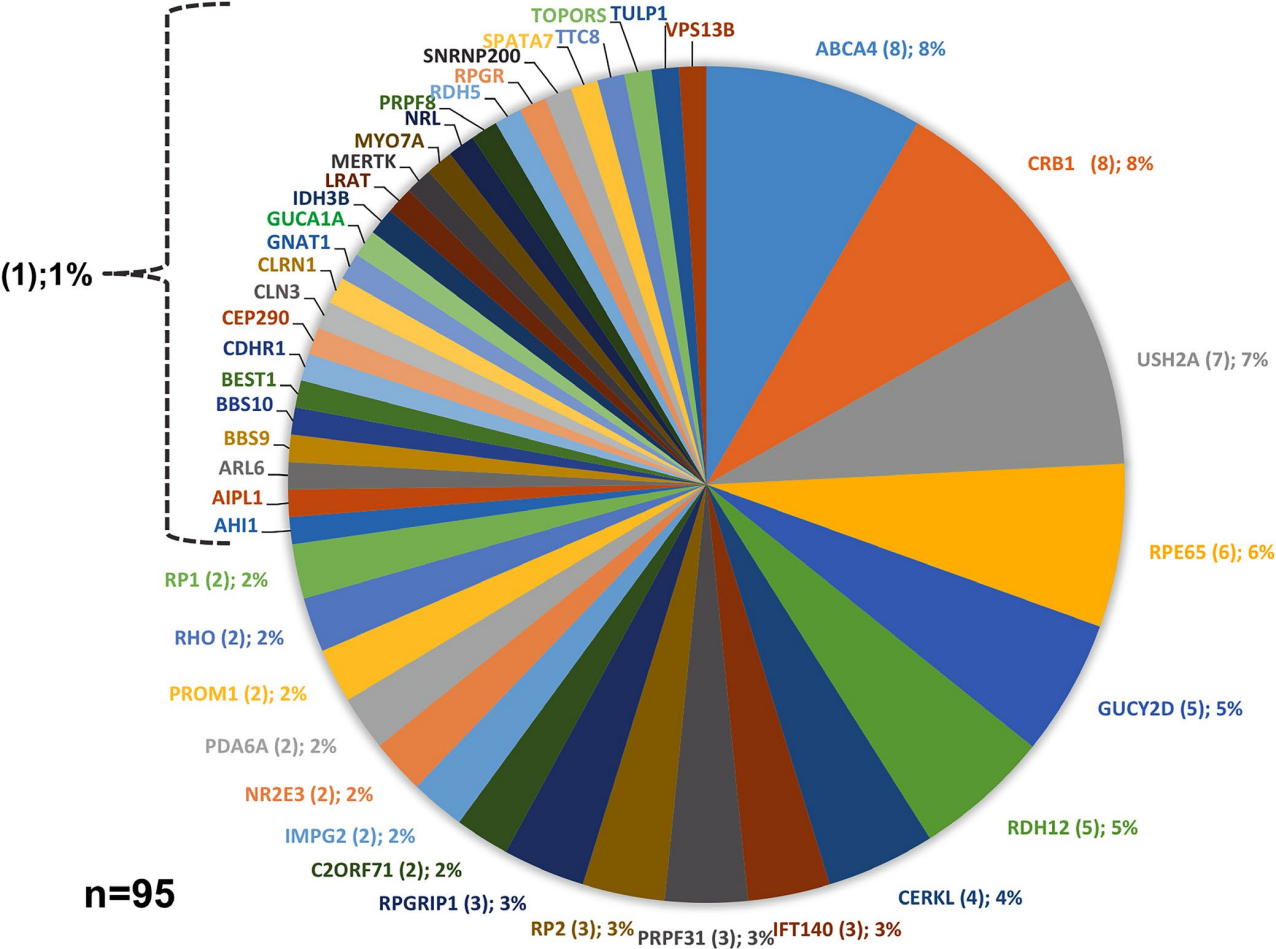
Mutational spectrum in 95 solved cases from our cohort. The number of cases carrying causative pathogenic variants is indicated below the name of the particular gene while the corresponding percentage is shown in parentheses

Pathogenic variants were identified in a total of 112 probands, accounting for a detection rate of 78%. In 95 cases, NGS allowed the identification of the causal pathogenic variant(s), reaching a solving rate of 66% (95/143). As detailed in Table 2, a total of 110 distinct pathogenic variants, including 53 novel ones (48%), were distributed among 44 different RD genes (Figure 1). Biallelic pathogenic variants were demonstrated in three quarters of solved RD cases from our study (Table 3). All novel variants identified in this study meet the criteria of the American College of Medical Genetics and Genomics (ACMG) to be classified as pathogenic or likely pathogenic variants (Richards et al., 2015; Table S2). All pathogenic and likely pathogenic novel variants were submitted to the Leiden Open Variation Database (LOVD; https://databases.lovd.nl/shared; Patient IDs: 00240417‐00240468). Missense changes were the most common type of identified variants (49%; 54/110), followed by frameshifts (25%; 28/110), nonsense (15%; 16/110), splicing (7%; 8/110), and CNVs (2%; 2/110). In addition, one start‐lost variant (1%) and one deep‐intronic variant (1%) were detected (Table 3). As shown in Figure 1, *ABCA4* (MIM *601691) and *CRB1 (*MIM *604210*)* were the most commonly involved genes in the group of solved cases (8%; 8/95 each), followed by *USH2A* (MIM *608400; 7%; 7/95), *RPE65* (MIM *180069) 6% (6/95), *GUCY2D* (MIM *600179; 5%; 5/95), and *RDH12* (MIM *608830; 5%; 5/95). As indicated in Table 4, different rates of molecular resolution were reached among the different RD subtypes. Selected examples of retinal phenotypes associated with particular pathogenic variants are shown in Figure S1.

**Table 2:** Causal genetic variations in the 95 solved cases from our cohort

Patient ID	Gene	NM ID	Genotype	cDNA change	Protein change	Reference^c^
*Retinitis Pigmentosa (AR)*						
3,566	*ABCA4*	NM_000350.2	Homozygous	c.4919G>A	p.Arg1640Gln	1
1,521	*ARL6*	NM_032146.4	Homozygous	c.373dupA	p.Ile125AsnfsTer7	NOVEL
3,356	*PCARE*	NM_001029883.2	Homozygous	c.947delA	p.Asn316MetfsTer7	2
3,520	*PCARE*	NM_001029883.2	Homozygous	c.947delA	p.Asn316MetfsTer7	2
1,977	*CDHR1*	NM_033100.3	Heterozygous	c.963G>C	p.Gln321His	NOVEL (ns)
			Heterozygous	c.2041−2A>C		NOVEL (ns)
2,792	*CERKL*	NM_001030311.2	Homozygous	c.1633_1636dupATCA	p.Ser546AsnfsTer21	NOVEL
2,741	*CERKL*	NM_001030311.2	Homozygous	c.847C>T	p.Arg283Ter	3
2,699	*CERKL*	NM_001030311.2	Heterozygous	c.424_427delAATT	p.Asn142Ter	NOVEL
			Heterozygous	c.1032_1039dupTGGGTTCT	p.Ser347LeufsTer77	NOVEL
3,919	*CLN3*	NM_001042432.1	Homozygous	c.266G>A	p.Arg89Gln	NOVEL
3,793	*CRB1*	NM_201253.2	Homozygous	c.2290C>T	p.Arg764Cys	4
1,853	*CRB1*	NM_201253.2	Homozygous	c.1125C>G	p.Tyr375Ter	5
3,662	*GNAT1*	NM_000172.3	Homozygous	c.282delT	p.Ala95HisfsTer9	NOVEL
1,830	*IFT140*	NM_014714.3	Heterozygous	c.1451C>T	p.Thr484Met	6
			Heterozygous	c.2786delC	p.Thr929SerfsTer21	NOVEL
3,332	*IMPG2*	NM_016247.3	Heterozygous	c.3093_3097dupTGGAG	p.Glu1033ValfsTer13	NOVEL
			Heterozygous	c.2038delG	p.Glu680SerfsTer21	NOVEL
1,140	*MERTK*	NM_006343.2	Homozygous	c.2531G>A	p.Arg844His	NOVEL
3,635	*PDE6A*	NM_000440.2	Homozygous	c.2302G>T	p.Glu768Ter	NOVEL
EC10	*PDE6A*	NM_000440.2	Heterozygous	c. 1705 C>A	p. Gln569Lys	7
			Heterozygous	c.1684 C>T	p.Arg562Trp	8
1,928^a^	*RDH5*	NM_002905.3	Homozygous	c.839G>A	p.Arg280His	9
3,777	*RDH12*	NM_152443.2	Homozygous	c.446T>C	p.Leu149Pro	10
2,884	*RDH12*	NM_152443.2	Homozygous	c.295C>A	p.Leu99Ile	11
872	*RDH12*	NM_152443.2	Heterozygous	c.295C>A	p.Leu99Ile	11
			Heterozygous	c.446T>C	p.Leu149Pro	10
2,637	*RP2*	NM_006915.2	Hemizygous	NC_000023.10 (NM_006915.2):c.(?_−1)_(768+1_769−1)del (exon 1–2 deletion)		NOVEL
3,354	*RP2*	NM_006915.2	Hemizygous	c.969+2T>G		NOVEL
3,544	*RPE65*	NM_000329.2	Homozygous	c.405T>A	p.Asn135Lys	NOVEL
169	*SPATA7*	NM_018418.4	Homozygous	c.322C>T	p.Arg108Ter	12
2,666	*USH2A*	NM_206933.2	Homozygous	c.11387C>T	p.Pro3796Leu	NOVEL
1,180	*USH2A*	NM_206933.2	Heterozygous	c.907C>A	p.Arg303Ser	13
			Heterozygous	c.5218delA	p.Ile1740PhefsTer10	NOVEL
2,822	*USH2A*	NM_206933.2	Heterozygous	c.2332G>T	p.Asp778Tyr	14
			Heterozygous	c.5836 C>T	p.Arg1946Ter	15
4,066	*USH2A*	NM_206933.2	Heterozygous	c.11156 G>A	p.Arg3719His	16
			Heterozygous	c.13348 C>T	p.Pro4450Ser	NOVEL
*Retinitis Pigmentosa (AD)*						
497	*NR2E3*	NM_014249.3	Heterozygous	c.166G>A	p.Gly56Arg	17
3,672	*NR2E3*	NM_014249.3	Heterozygous	c.166G>A	p.Gly56Arg	17
1,031	*NRL*	NM_006177.3	Heterozygous	c.148 T>C	p.Ser50Pro	18
3,650	*PRPF8*	NM_006445.3	Heterozygous	c.6928 A>G	p.Arg2310Gly	19
3,596	*PRPF31*	NM_015629.3	Heterozygous	c.682G>C	p.Ala228Pro	20
3,542	*PRPF31*	NM_015629.3	Heterozygous	c.866_879delGGAAAGCGGCCCGG	p.Arg289ProfsTer30	21
4,013	*PRPF31*	NM_015629.3	Heterozygous	c.866_879delGGAAAGCGGCCCGG	p.Arg289ProfsTer30	21
3,627	*RHO*	NM_000539.3	Heterozygous	c.491C>A	p.Ala164Glu	22
3,065	*RHO*	NM_000539.3	Heterozygous	c.557C>G	p.Ser186Trp	23
3,583	*RP1*	NM_006269.1	Heterozygous	c.2029C>T	p.Arg677Ter	24
EC17	*TOPORS*	NM_005802.4	Heterozygous	c.2554_2557delGAGA	p.Glu852GlnfsTer13	25
*Simplex RP*						
3,632	*ABCA4*	NM_000350.2	Heterozygous	c.1417_1420dupATTA	p.Thr474AsnfsTer4	NOVEL
			Heterozygous	c.5196+1G>A		26
EC09	*CERKL*	NM_001030311.2	Homozygous	c.847C>T	p.Arg283Ter	3
3,868	*IDH3B*	NM_006899.3	Homozygous	c.857G>A	p.Gly286Glu	NOVEL
EC18	*IFT140*	NM_014714.3	Heterozygous	c.386T>G	p.Leu129Trp	27
			Heterozygous	c.1377G>A	p.Trp459Ter	28
3,343	*RDH12*	NM_152443.2	Homozygous	c.295C>A	p.Leu99Ile	11
3,647	*RDH12*	NM_152443.2	Heterozygous	c.295C>A	p.Leu99Ile	11
			Heterozygous	c.697G>C	p.Val233Leu	29
3,527	*RP1*	NM_006269.1	Homozygous	c.3150delA	p.Lys1050AsnfsTer7	NOVEL
3,751	*RPE65*	NM_000329.2	Heterozygous	c.131G>A	p.Arg44Gln	30
			Heterozygous	c.61delG	p.Glu21AsnfsTer10	NOVEL
3,261	*RPE65*	NM_000329.2	Heterozygous	c.386 C>T	p.Thr129Ile	NOVEL
			Heterozygous	c.1067dupA	p.Asn356LysfsTer9	31
3,524	*RPE65*	NM_000329.2	Homozygous	c.95−2A>T		32
3,340	*RPGR*	NM_001034853.1	Hemizygous	c.1859_1860delAG	p.Lys620ArgfsTer9	NOVEL
3,275	*RPGRIP1*	NM_020366.3	Homozygous	c.154C>T	p.Arg52Ter	33
3,268	*USH2A*	NM_206933.2	Heterozygous	c.12575G>A	p.Arg4192His	34
			Heterozygous	c.3629T>C	p.Leu1210Pro	NOVEL
4,020	*SNRNP200*	NM_014014.4	Heterozygous	c.3260C>T	p.Ser1087Leu	35
*Leber Congenital Amaurosis*						
438	*AIPL1*	NM_014336.3	Homozygous	c.547G>T	p.Gly183Ter	36
3,480	*CRB1*	NM_201253.2	Heterozygous	c.613_619delATAGGAA	p.Ile205AspfsTer13	37
			Heterozygous	c.2797T>C	p.Cys933Arg	NOVEL
2,712	*CRB1*	NM_201253.2	Homozygous	c.3014A>T	p.Asp1005Val	38
1,97	*CRB1*	NM_201253.2	Heterozygous	c.1125C>G	p.Tyr375Ter	5 (ns)
			Heterozygous	c.3158T>A	p.Met1053Lys	NOVEL (ns)
3,043	*CRB1*	NM_201253.2	Heterozygous	c.3822C>A	p.Cys1274Ter	NOVEL (ns)
			Heterozygous	c.2290C>T	p.Arg764Cys	4 (ns)
2,257	*GUCY2D*	NM_000180.3	Homozygous	c.1157delA	p.Gln386ArgfsTer9	NOVEL
1,985	*GUCY2D*	NM_000180.3	Heterozygous	c.982G>C	p.Ala328Pro	NOVEL
			Heterozygous	c.997G>A	p.Glu333Lys	NOVEL
3,961	*GUCY2D*	NM_000180.3	Homozygous	c.914delA	p.His305ProfsTer90	39
2,566	*GUCY2D*	NM_000180.3	Homozygous	c.389delC	p.Pro130LeufsTer36	40
1,274	*LRAT*	NM_004744.3	Homozygous	c.614_615delCT	p.Ser205TyrfsTer51	NOVEL
3,448	*RPE65*	NM_000329.2	Homozygous	c.370C>T	p.Arg124Ter	41
2,934	*RPE65*	NM_000329.2	Heterozygous	c.190delC	Gln64LysfsTer30	NOVEL
			Heterozygous	c.11+5G>A		42
3,483	*RPGRIP1*	NM_020366.3	Homozygous	c.1624delG	p.Ala542GlnfsTer2	NOVEL
3,592	*RPGRIP1*	NM_020366.3	Homozygous	c.1116delA	p.Lys372AsnfsTer3	NOVEL
2,669	*TULP1*	NM_003322.3	Homozygous	c.1102G>A	p.Gly368Arg	NOVEL
*Cone‐Rod Dystrophy*						
3,585	*ABCA4*	NM_000350.2	Heterozygous	c.4919G>A	p.Arg1640Gln	1 (ns)
			Heterozygous	c.4854G>C	p.Trp1618Cys	NOVEL (ns)
1,175	*ABCA4*	NM_000350.2	Heterozygous	c. 6221G>T	p.Gly2074Val	43
			Heterozygous	c.6282+3A>T		NOVEL
3,904	*CRB1*	NM_201253.2	Homozygous	c.936T>G	p.Asn312Lys	44
3,267	*CRB1*	NM_201253.2	Homozygous	c.2506C>A	p. Pro836Thr	45
2,996	*GUCA1A*	NM_000409.3	Heterozygous	c.328_337delGATGAGCTGC	p.Asp110SerfsTer18	NOVEL
3,525	*GUCY2D*	NM_000180.3	Heterozygous	c.2705T>C	p.Val902Ala	NOVEL
*Macular Dystrophy*						
3,457	*ABCA4*	NM_000350.2	Homozygous	c.6148G>C	p.Val2050Leu	46
3,522	*ABCA4*	NM_000350.2	Heterozygous	c.5819T>C	p.Leu1940Pro	47
			Heterozygous	c.5324T>A	p.Ile1775Asn	48
3,529	*ABCA4*	NM_000350.2	Heterozygous	c.6221G>T	p.Gly2074Val	43 (ns)
			Heterozygous	c.5318C>T	p.Ala1773Val	48 (ns)
3,286	*ABCA4*	NM_000350.2	Heterozygous	c.3383A>G	p.Asp1128Gly	NOVEL (ns)
			Heterozygous	c.4804delA	p.Ile1602TyrfsTer8	NOVEL (ns)
3,109	*BEST1*	NM_001139443.1	Heterozygous	c.671A>G	p.Tyr224Cys	49^b^
3,068	*IFT140*	NM_014714.3	Homozygous	c.4252C>T	p.Gln1418Ter	NOVEL
1,602	*IMPG2*	NM_016247.3	Heterozygous	c.2887 A>G	p.Ser963Gly	NOVEL
EC07	*PROM1*	NM_006017.2	Heterozygous	c.1117C>T	p.Arg373Cys	50
4,079	*PROM1*	NM_006017.2	Heterozygous	c.1117C>T	p.Arg373Cys	50
*Syndromic Retinal Dystrophy (AR)*						
3,602	*AHI1*	NM_017651.4	Homozygous	c.2029 A>C	p. Thr677Pro	NOVEL
2,831	*BBS9*	NM_198428.2/ NC_000007.14	Homozygous	c.1329+1738C>T/ g.33346372C>T		NOVEL
2,405	*BBS10*	NM_024685.3	Homozygous	c.9_15delinsGC	p.Ser3ArgfsTer91	51
3,531	*MYO7A*	NM_000260.3	Homozygous	c.(2,282+1_2283−1_(2,904+1_2905−1)del (exon 20–23 deletion)		NOVEL
2,583	*TTC8*	NM_144596.2	Homozygous	c.674G>A	p.Trp225Ter	NOVEL
3,776	*USH2A*	NM_206933.2	Homozygous	c.11389+1G>A		NOVEL
3,723	*VPS13B*	NM_017890.4	Heterozygous	c.5086 C>T	p. Arg1696Ter	52
			Heterozygous	c.8978 A>G	p.Asn2993Ser	53
*Syndromic Retinal Dystrophy (Simplex cases)*						
3,593	*CEP290*	NM_025114.3	Homozygous	c.2605 C>T	p.Gln869Ter	NOVEL
3,436	*CLRN1*	NM_001195794.1	Heterozygous	c.189C>A	p. Tyr63Ter	54
			Heterozygous	c.41 G>A	p.Gly14Glu	NOVEL
3,781	*USH2A*	NM_206933.2	Homozygous	c.1841−2A>G		55
X‐Linked RP						
3,533	*RP2*	NM_006915.2	Hemizygous	c.1A>G	p.Met1?	NOVEL

Note

One hundred and ten different pathogenic variants were detected within the 95 solved cases in our cohort, including 53 previously undescribed changes. All novel variants identified in this study meet the criteria of the American College of Medical Genetics and Genomics (ACMG) to be classified as pathogenic or likely pathogenic variants (Table S2). All cases underwent familial segregation analysis except in six probands whose variants are marked as (ns).

a Cases diagnosed as fundus albipunctatus with an autosomal recessive pattern.

b This variant was previously reported as p.Tyr284Cys.

c References are provided in Supplementary references for Table 2.

**Table 3:** Frequencies and zygosity of the different types of variants identified in the 95 solved cases from our cohort

Type of variants	# of identified variants	Percentage
Missense	54	49
Frameshift	28	25
Nonsense	16	15
Splicing	8	7
CNV	2	2
Start‐Lost	1	1
Deep‐intronic	1	1
Total	110	100

Zygosity	# of cases	Percentage
Homozygous	46	49
Compound heterozygous	27	28
Heterozygous	18	19
Hemizygous	4	4
Total	95	100

**Table 4:** Molecular solving rates by RD subtype in this study

Type of RD	Total cases	Solved cases	Solving rate	Most commonly mutated genes
ARRP	37	29	78%	*USH2A* (4 cases), *CERKL* (3 cases), *RDH12* (3 cases)
ADRP	16	11	69%	*PRPF31* (3 cases)
Simplex RP	29	14	48%	*RPE65* (3 cases)
LCA	21	15	71%	*CRB1* (4 cases), *GUCY2D* (4 cases)
CRD	7	6	86%	*ABCA4* (2 cases), *CRB1* (2 cases)
MD/STGD	11	9	82%	*ABCA4* (4 cases)
Syndromic ARRD	12	7	58%	—
Simplex syndromic RD	7	3	43%	—
X‐linked RP	3	1	33%	—
Total	143	95	66%	

Abbreviations: ADRP, Autosomal Dominant Retinitis Pigmentosa; ARRD, Autosomal Recessive Retinal Dystrophy; ARRP, Autosomal Recessive Retinitis Pigmentosa; CRD, Cone‐Rod Dystrophy; LCA, Leber Congenital Amaurosis; MD, Macular Disease; RD, Retinal Dystrophy; RP, Retinitis Pigmentosa; STGD, Stargardt Disease.

### 3.4 Molecular findings in patients classified as autosomal recessive RP by genealogy

Targeted NGS allowed us to solve 78% (29/37) of ARRP cases with *USH2A* being the most commonly involved gene (four cases; Table 4). Novel pathogenic variants were identified in *ARL6* (MIM *180069; p.Ile125Asnfs*7) and *GNAT1* (MIM *139330; p.Ala95Hisfs*9; Table 2), confirming the recently suggested involvement of these genes in non‐syndromic RP (Aldahmesh et al., 2009; Méjécase et al., 2016). Of note, the identification of a novel two‐exon deletion (Figure S2a–c) and of a novel splicing variant in *RP2* (MIM *300757) in two cases from this group allowed their reclassification to XLRP. In two ARRP subjects carrying biallelic pathogenic variants in *USH2A*, clinical reassessment disclosed mild hypoacusia and these cases are under specialized evaluation for their possible reclassification to Usher syndrome.

### 3.5 Molecular findings in patients with autosomal dominant RP

Eleven of 16 cases (69%) with an apparent autosomal dominant pattern were molecularly solved. A total of nine different pathogenic variants distributed among seven different genes were demonstrated, including previously reported missense changes in *NR2E3* (MIM *604485), *NRL* (MIM *162080), *PRPF8* (MIM *607300), *PRPF31* (MIM *606419), and *RHO* (MIM *180380), and previously described truncating variants in *RP1* (MIM *603937) and *TOPORS* (MIM *609507; Table 2).

### 3.6 Molecular findings in subjects with simplex retinitis pigmentosa

Pathogenic variants were recognized in 48% (14/29) of simplex RP cases from our cohort. Of these, 86% (12/14) of patients were reclassified as autosomal recessive cases based on molecular findings, 7% (1/14) was shown to carry causative variations in X‐linked genes, while one (7%) was reclassified as autosomal dominant. Variants in *RPE65* were the most prevalent defect in this group occurring in 21% (3/14) of probands, followed by *RDH12* pathogenic variants in 14% (2/14) of individuals (Table 2). Additionally, a novel homozygous missense change (p.Gly286Glu) in IDH3B (MIM *604526) was detected in one case, supporting its involvement in ARRP.

### 3.7 Molecular findings in patients with Leber congenital amaurosis

For patients with LCA, clear pathogenic variants were identified in 71% (15/21). The most commonly involved genes in this group were *CRB1* and *GUCY2D*, each harboring biallelic defects in 27% (4/15) of probands, followed by *RPE65* and *RPGRIP1* (MIM *605446), each with pathogenic variants in 13% (2/15) of solved LCA cases (Table 2).

### 3.8 Molecular findings in subjects with Cone‐Rod Dystrophy and Macular dystrophy/Stargardt disease

Seven cases were classified as CRD, while 11 were categorized as MD/STGD. Pathogenic variants were identified in 86% (six of seven) CRD cases, including biallelic *ABCA4* (two cases) and *CRB1* (two cases) defects (Table 4). The other two CRD solved cases carried heterozygous variants in AD genes, including a novel truncating variant in GUCA1A (MIM *600364; p.Asp110SerfsTer18) and a novel p.Val902Ala replacement in GUCY2D. In cases clinically classified as MD/STGD, pathogenic variants were demonstrated in 82% (nine of 11) of the probands, with most causal variants (4/9) being localized at *ABCA4*. In an additional familial MD/STGD case, a novel IFT140 (MIM *614620) truncating defect (p.Gln1418Ter) was demonstrated (Table 2).

### 3.9 Molecular findings in patients with X‐linked retinitis pigmentosa

According to pedigree structure, three XLRP cases were included in this study. In one pedigree, a novel start‐lost variant was demonstrated in *RP2* (c.1A>G; p.Met1?). No candidate variants were identified in the other two pedigrees.

### 3.10 Molecular findings in patients with syndromic retinal dystrophies

Twelve AR syndromic and seven simplex syndromic RD cases, representing 8% and 5% of the total of patients, respectively, were genotyped. The most common syndromic RDs were Usher (*n* = 8) and Bardet‐Biedl (*n* = 5) syndromes. In addition, cases of Joubert syndrome (*n* = 2), Cohen syndrome (*n* = 1), and undiagnosed syndromic RD (*n* = 3) were also ascertained. For syndromic ARRD cases, a solving rate of 58% (7/12) was achieved, while for simplex syndromic RD cases, the solving rate was of 43% (3/7). In a familial case of Usher syndrome, a novel *MYO7A* (MIM *276903) intragenic deletion encompassing exons 20–23 was identified (Figure S2d–f).

**Figure 2:**
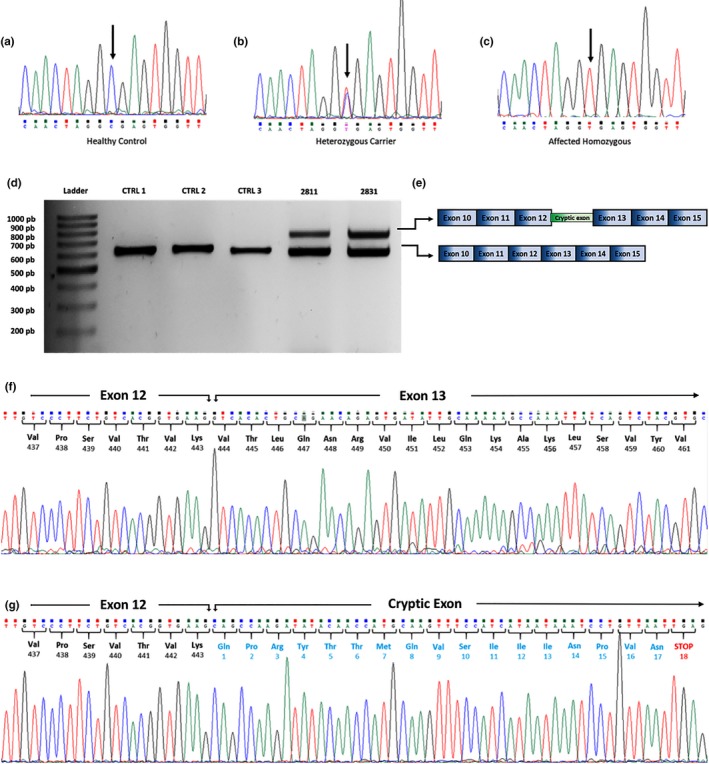
RNA analysis of a deep‐intronic BBS9 variant in a familial case of Bardet‐Biedl syndrome. Genomic sequence of intron 12–13 showing the BBS9 c.1329+1738C>T (NC_000007.14:g.33346372C>T) variant in a healthy homozygous control (a), a heterozygous carrier parent (b), and an affected homozygous patient (c). Arrows indicate the change. (d) RT‐PCR products from three healthy controls (CTRL 1–3) and two siblings (2,811 and 2,831) affected with Bardet–Biedl syndrome. BBS9 mRNA from affected siblings amplified two products, the shorter amplicon representing the normal BBS9 transcript spanning from exon 10 to exon 15 and a larger amplicon with a cryptic 144 bp exon retained between exons 12 and 13 (e). (f) Sanger sequencing of the BBS9 transcript showed the normal boundary sequence between exons 12 and 13. (g) Sanger sequencing of the BBS9 aberrant transcript revealed a new cryptic exon retained after exon 12 with a premature stop codon 18 triplets downstream

In two siblings with Bardet‐Biedl syndrome, a deep‐intronic *BBS9* (MIM *607968) homozygous variant (NC_000007.14:g.33346372C>T; NM_198428.2: c.1329+1738C>T) was recognized. In silico analyses using five different splicing algorithms predicted that this variant could activate a new donor site (Table S3). This *BBS9* variant was absent from public population databases and from more than 100 in‐house exomes, and was identified in the heterozygous state in parental DNA (Figure 2a–c). This *BBS9* variant was analyzed at RNA level to support their involvement in the disease (see below).

### 3.11 RNA‐Splicing effect of a BBS9 deep‐intronic variant (c.1329+1738C>T)

A homozygous c.1329+1738C>T variant was identified in two siblings affected by Bardet‐Biedl syndrome. RT‐PCR performed on RNA isolated from saliva of both patients revealed the presence of an aberrant BBS9 splicing product, in addition to the expected amplicon (Figure 2d). The aberrant splicing product was absent in healthy controls (Figure 2d). Sequencing of the larger product confirmed the insertion of a 144 bp pseudoexon between *BBS9* exons 12 and 13 (Figure 2e), introducing a premature stop codon 18 triplets downstream (Figure 2f,g).

### 3.12 Unsolved cases

Forty‐eight (34%) RD cases from our cohort remained unsolved after NGS, including eight ARRP, five AD RP, 15 simplex RP, six LCA, one CRD, two MD/STGD, five syndromic ARRD, four simplex syndromic RD, and two apparently XLRP cases. As detailed in Table S3, 25 heterozygous variants in 18 different recessive RD genes were detected in 17 of the 43 unsolved probands. Nine of the identified changes were previously reported as pathogenic, while most of the remaining were predicted to be pathogenic by in silico analysis, had a MAF of <5% in population databases, or were frameshift or nonsense variants that predicted truncated proteins. Five of 25 identified changes (20%) occurred in *USH2A*, which was the most commonly affected gene in this group of unsolved patients (Table S4). Furthermore, seven variants of uncertain significance (VUS) were found in four cases from this group. Although these variants were detected in genes known to be involved in different types of RDs, they show ACMG criteria for both benign and pathogenic classification. Therefore, these variants were classified as VUS and could not be considered as causal variants for their respective cases (Table S5).

### 3.13 Genetic screening in relatives

From the 95 solved cases, a total of 258 first‐ or second‐degree relatives were tested for the respective pathogenic variant(s), allowing the identification of 124 autosomal or X‐linked carriers (Table S6). In addition, pathogenic variants were confirmed in 98 affected relatives, while 33 subjects were proven to be wild‐type homozygotes. Familial genetic analysis in compound heterozygous cases confirmed the *trans* configuration of variants, except in six instances where no first‐degree relatives were available (Table 2).

## 4 DISCUSSION

In this work, the results of NGS screening in a large cohort of Mexican individuals with distinct types of RDs are presented. Of a total of 143 unrelated probands, causal variants were demonstrated in 95 cases, for a molecular diagnosis rate of 66%. This study represents the largest cohort of molecularly analyzed RD subjects from Mexico and Latin America, and our results demonstrate extensive genic and allelic heterogeneity underlying these disorders in our population. As it has been shown in previous studies (Bernardis et al., 2016; Bravo‐Gil et al., 2016; Glöckle et al., 2014; Patel et al., 2016), the molecular diagnostic yield varied among subtypes of RD in our cohort, with CRD (86% solving rate), ARRP (78%), MD (82%), and LCA (71%) being the commonest molecularly solved disorders. In contrast, RD groups with lowest rates of molecular diagnosis were simplex RP and AD RP, with 48% and 69%, respectively (Table 4). Our findings are in agreement with previously published series that showed high molecular diagnostic yields for ARRP and LCA and lower rates for AD and simplex RP (Bernardis et al., 2016; Bravo‐Gil et al., 2016; Glöckle et al., 2014; Patel et al., 2016).

An interesting finding in our study was the high rate of novel variants in RD genes, illustrating the value of molecularly testing different populations. The recognition of novel pathogenic variants in RD is warranted as it allows a better characterization of the mutational landscape resulting in retinal degeneration, permits refinement of genotype–phenotype correlations, and identify ethnic‐specific founder effects. In our cohort, 48% (53 of 110) of pathogenic variants were novel and most of them predicted missense, frameshift, and nonsense alterations. Our identified rate of novel pathogenic variants in RD genes is similar to that observed in several series, ranging from 40% to 50% (Ge et al., 2015; Patel et al., 2016; Zhao et al., 2015). Genes with most novel defects in the present study were *ABCA4*, *USH2A*, *RPE65*, and *GUCY2D*. *ABCA4* has been demonstrated as the most frequently involved gene in several RD cohorts recently analyzed by NGS (Carrigan, Duignan, Malone, et al., 2016). It could be anticipated that the percentage of novel deleterious changes identified in individual cohorts will decrease as additional groups of RD patients from different ethnic backgrounds be molecularly analyzed.

In our work, several variants were demonstrated in genes rarely associated with RDs, supporting their involvement in retinal disease. In 2008, pathogenic variants in *IDH3B* were identified in two ARRP cases from North America (Hartong et al., 2008) with no additional confirmatory cases published since then. Here, a homozygous (p.Gly286Glu) IDH3B variant was demonstrated in a simplex RP case. Previously identified *IDH3B*‐related RP cases carried the homozygous p.Ile163fs and p.Leu98Pro changes (Hartong et al., 2008). Thus, to the best of our knowledge, the patient described here is the third published RP case arising from biallelic *IDH3B* variants. As reported in a previous *IDH3B‐*linked RP case, early onset subcapsular cataracts developed in our patient, suggesting an incipient genotype–phenotype correlation for IDH3B‐related RD. IDH3B catalyzes the oxidative decarboxylation of isocitrate to produce α‐ketoglutarate while converting nicotine adenine dinucleotide (NAD+) to nicotinamide adenine dinucleotide Hydrogenated (NADH) in the Krebs cycle (Hartong et al., 2008). ARL6 is a member of the ARF‐like family of small GTPases, with a predicted function in membrane trafficking at the base of the ciliary organelle. Biallelic mutations in *ARL6* result in Bardet‐Biedl syndrome type 3 (BBS3; Fan et al., 2004). Previously, a consanguineous family segregating AR non‐syndromic RP due to a homozygous p.Ala89Val ARL6 variant was identified by Aldahmesh et al. (2009). The identification in the present work of a novel homozygous p.Ile125Asnfs*7 truncating ARL6 variant in a non‐syndromic RD patient supports that particular defects in this gene can result exclusively in retinal damage. The patient described here was born from an endogamous marriage and had two affected sibs aged 25 and 14 years. All three affected sibs complained of blurred vision and nyctalopia, both starting by the age of 4 years. Funduscopic examination of the propositus at the age of 14 years showed pale optic discs, retinal vessel attenuation, and salt‐and‐pepper retinopathy. Bone spicules‐like retinal pigmentation was not observed. Developmental milestones were according to age and no intellectual disability was present.

Defects in genes usually associated with syndromic retinal disease are increasingly found to cause non‐syndromic inherited retinal degenerations. For example, pathogenic changes in *CLN3* (MIM *607042) are well known as causative of the severe disease juvenile neuronal ceroid lipofuscinosis or Batten disease, a rare neurodegenerative disorder associating early retinal degeneration and progressive neurologic deterioration (Jalanko & Braulke, 2009). Nonetheless, *CLN3* variants were recently identified in patients with non‐syndromic RDs (Ku et al., 2017; Wang et al., 2014). In our study, a novel homozygous p.Arg89Gln CLN3 substitution was identified in an ARRP patient and in her affected sister. Of note, nonsense variants at CLN3 arginine 89 have been previously demonstrated in two unrelated Batten disease patients (Kousi, Lehesjoki, & Mole, 2012; Pérez‐Poyato et al., 2011). However, no apparent genotype–phenotype correlation currently exists in *CLN3*‐related non‐syndromic RD. Non‐syndromic *CLN3*‐retinal phenotype includes both adult and early onset phenotype, mild nyctalopia, variable loss of visual acuity, and rod‐cone dystrophy with marked diminished rod function and significant but variable cone system dysfunction (Ku et al., 2017).

*GNAT1* encodes the alpha subunit of the rod protein complex called transducin. Pathogenic alterations in this gene have been typically associated with both AD and AR congenital stationary night blindness (CSNB; Dryja, Hahn, Reboul, & Arnaud, 1996; Naeem et al., 2012). Recently, homozygous truncating *GNAT1* changes (p.Cys321* and p.Gln302*) were identified in two sporadic patients with rod‐cone dystrophy (Carrigan, Duignan, Humphries, et al., 2016; Méjécase et al., 2016). In our work, a homozygous frameshifting variant was demonstrated in a RP familial case, confirming that truncating defects in *GNAT1* results in the more severe rod‐cone dystrophy phenotype and providing the first description of a familial *GNAT1‐*linked RP case. Available data indicate that while dominant and recessive forms of CSNB are associated with missense *GNAT1* variants, C‐terminal nonsense variants manifest as rod‐cone dystrophy.

In two brothers with Bardet‐Biedl syndrome, a deep‐intronic *BBS9* homozygous variant, c.1329+1738C>T (transcript NM_198428.2), was recognized. RNA analysis demonstrated that this variant causes aberrant splicing products by activation of a cryptic exon between exons 12 and 13 in the BBS9 mRNA. To the best of our knowledge, this is the first time that such mechanism is described for BBS9‐related Bardet‐Biedl syndrome. Interestingly, in addition to the aberrantly spliced *BBS9* product, a normally spliced *BBS9* transcript was demonstrated in RNA from saliva cells from these siblings. The presence of wild‐type mRNA product in affected individuals carrying homozygous intronic variants has been previously observed for the *CEP290* c.2991+1655A>G pathogenic variant in LCA patients (den Hollander et al., 2006) and for the *ARL2BP* c.390+5G>A pathogenic variant in ARRP (Fiorentino et al., 2018). Recent evidence indicates that for the above mentioned *CEP290* variant there is a different ratio of aberrantly versus correctly spliced products in different cell types and that even within the retina, the differential inclusion of a pseudoexon could be a cell type‐specific event (Parfitt et al., 2016). A similar mechanism could be attributed to explain the BBS9 expression pattern in our patients. At the clinical level, both patients exhibited RD, obesity, polydactyly, and intellectual disability with no apparent difference in phenotypic severity.

In our study, 48% (14/29) of RP simplex or sporadic cases were solved, allowing for the precise definition of the inheritance pattern in such individuals. Our findings are in accordance with several series, where AR genes were the most commonly mutated among subjects with nonfamilial RP (Bravo‐Gil et al., 2017; De Castro‐Miró et al., 2016; Neveling et al., 2012).

Inconsistency between assigned inheritance patterns and the genetic test results is commonly observed in RD NGS studies (Chen et al., 2018; Jones et al., 2017). The precise identification of inheritance pattern by means of the genetic results allows for accurate genetic counseling. As an example, from a sibship of six females and six males in a family included in our study, four males were affected by Usher syndrome, suggesting an X‐linked recessive transmission of disease; however, NGS genotyping efficiently identified a homozygous pathogenic variant in *USH2A*, allowing a redefinition of the inheritance pattern as autosomal recessive. In a family with a presumptive case of sporadic CRD, the identification of a heterozygous frameshift variant in the dominant *GUCA1A* gene led us to the identification of lack of penetrance in the transmitting father.

In two young brothers referred with syndromic RD and with a history of neonatal hypotonia, microcephaly, and facial dysmorphism, the identification of biallelic pathogenic variants in the *VPS13B* (MIM *216550) allowed a final diagnosis of Cohen syndrome. These patients underwent specialized hematologic, cardiologic, and neurologic assessment for early recognition of associated complications.

NGS has emerged as a powerful tool for identification of the molecular defect in RDs. Among the benefits of causal variant recognition, the possibility of identifying candidate patients for novel treatments is central. This is particularly important for individuals with *RPE65*‐related RD, who can benefit from a recently commercially approved gene therapy to restore vision (Ameri, 2018). In our series, a total of six (four RP and two LCA) nonrelated probands were found to harbor biallelic changes in *RPE65*. In our study, a total of 12 individuals (including probands and affected relatives in familial cases) can be considered as candidates to receive *RPE65* therapy for their disease.

Although no major founder mutation effects were identified in our RD cohort, two identical variants were recognized several times in apparently unrelated families. The first variant was p.Arg289Profs*30 in PRPF31, that was identified in two families segregating autosomal dominant RP. Both families originated from the Yucatan peninsula, the same region of origin of a recently published AD RP family carrying the same *PRPF31* variant (Villanueva et al., 2014). The second recurrent variant occurred in *RDH12* and consisted in p.Leu99Ile, which accounted for six of 10 *RDH12* pathogenic alleles identified in this work.

Carrier detection for recessive genetic conditions is extremely useful, especially in countries like Mexico in which endogamy and consanguineous marriage are still common in some geographic areas. In our study, a total of 73 causal genotypes were identified in subjects with recessive (including RP, LCA, STGD, and MD) or simplex RDs. From these families, a total of 194 first‐ or second‐degree relatives were tested for the respective pathogenic variant, allowing the identification of 120 heterozygous carriers, 53 subjects with biallelic defects (homozygous or compound heterozygous), and 21 wild‐type homozygotes (Table S4). As a consequence, genetic counselling and reproductive decisions in these families are being greatly aided. Another important aspect of genetic familial screening is the possibility to confirm the *trans* configuration of variants in compound heterozygous cases. For instance, in a familial ARRP case, we identified three apparently pathogenic variants in *EYS* (c.3443+1G>T; p.Thr1084Pro; p.Asp1468His). Interestingly, these variants have occurred together in at least one previously reported RP case, and had been considered as biallelic variants probably responsible for the disease (see Table S3 for references). However, familial segregation in our study identified that all three *EYS* variants were located in *cis* configuration, suggesting that these variants are, in fact, linked in a single haplotype.

Among the group of patients in which a final molecular diagnosis was not reached, 35% (17/48) had a single pathogenic allele in well‐known recessive RD genes. Focused analysis for additional pathogenic changes in the corresponding gene failed to identify the second causative variant, suggesting that it could be located in regions not screened by our panel (deep‐intronic or promoters) or that some cases could arise from digenic effects. In such cases, it is expected that in‐depth methodologies, as whole exome or whole genome sequencing, will be able to recognize the “second hit” or “missed” pathogenic variant (González‐del Pozo et al., 2018). While negative cases can be also explained by hitherto undescribed RD genes, it is important to consider that targeted NGS has certain technical limitations. In fact, a weakness in our study was the low coverage of adenine/guanine‐repetitive regions (46% the ORF 15 exon at an average read depth of 28X) of the X‐linked *RPGR* (MIM *312610). This limitation has been frequently reported in NGS of a number of RD cohorts (Huang, Wu, Lv, Zhang, & Jin, 2015; Stone et al., 2017), and ideally *RPGR* exon 15 must be analyzed by alternative methodologies (Chiang et al., 2018; Li et al., 2016). Another limitation of our work is that most deep‐intronic variants are not covered by our panel.This prevents detection of variants found deep within introns that could be associated to RDs, such as the recurrent pathogenic variant c.2991+1655A>G in *CEP290* that has commonly been associated to cases of LCA (MIM *610142; den Hollander et al., 2006).

## 5 CONCLUSIONS

In conclusion, our results contribute to expand the spectrum of gene defects causing human RDs, and illustrate the convenience of NGS for molecular diagnosis of these genetically heterogeneous diseases. A molecular diagnosis was achieved in 66% of patients and 53 novel RD‐causing pathogenic variants were recognized, allowing not only a more accurate clinical classification and prognosis in a number of cases, but also a well‐supported genetic counseling in affected families.

## CONFLICT OF INTEREST

The authors declare no potential conflict of interests.

## AUTHOR CONTRIBUTIONS

Josue Ronquillo, Agustin Rodas‐Serrano, Rodrigo Matsui, Rocio Arce‐Gonzalez, Federico Graue‐Wiechers, Mario Gutiérrez‐Paz, Tatiana Urrea‐Victoria, and Ulises de Dios Cuadras recruited the study participants and conducted the clinical evaluations. Juan C. Zenteno, Leopoldo A. Garcia‐Montaño, and Marisa Cruz‐Aguilar planned and organized the study and wrote the manuscript. Juan C. Zenteno, Leopoldo A. García‐Montaño, Marisa Aguilar‐Cruz, Carlos I. Vencedor‐Meraz, Luis Aguilar‐Castul, and Oscar F. Chacon‐Camacho acquired, analyzed, and interpreted the data. All authors have approved the final manuscript.
