# Supplementary material for: Extensive genic and allelic heterogeneity underlying inherited retinal dystrophies in Mexican patients molecularly analyzed by next‐generation sequencing
Source: Mol Genet Genomic Med. 2019 Nov 17;8(1):10.1002/mgg3.1044. doi: 10.1002/mgg3.1044 (PMC6978239; doi:10.1002/mgg3.1044)
Supplement: Supplementary file 1 [file MGG3-8-0-s001.pdf]

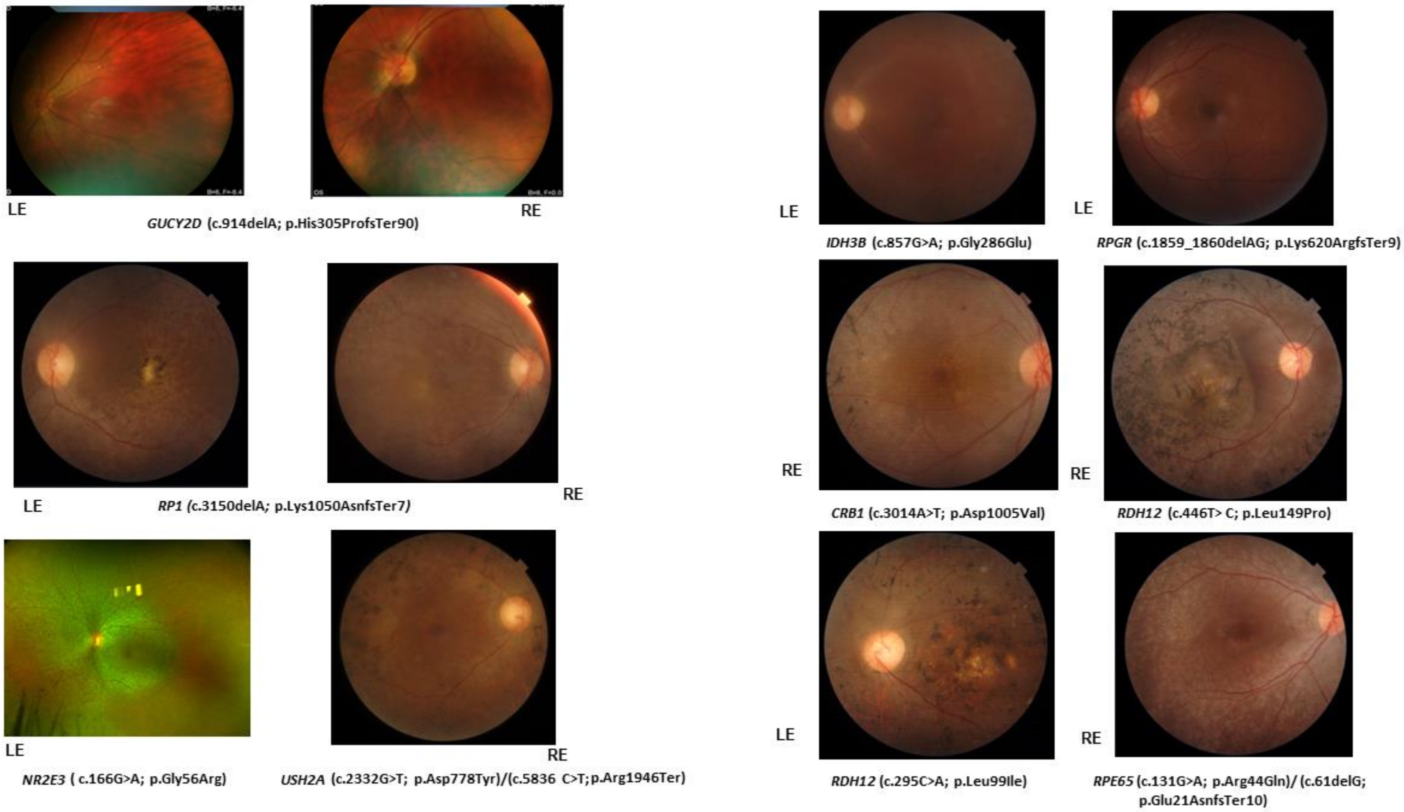

Supp. Figure S1.- Selected retinal phenotypes in RD patients from our cohort. The identified pathogenic variant is indicated in each case. For specific diagnosis see table 2. **LE**: left eye; **RE**: right eye.

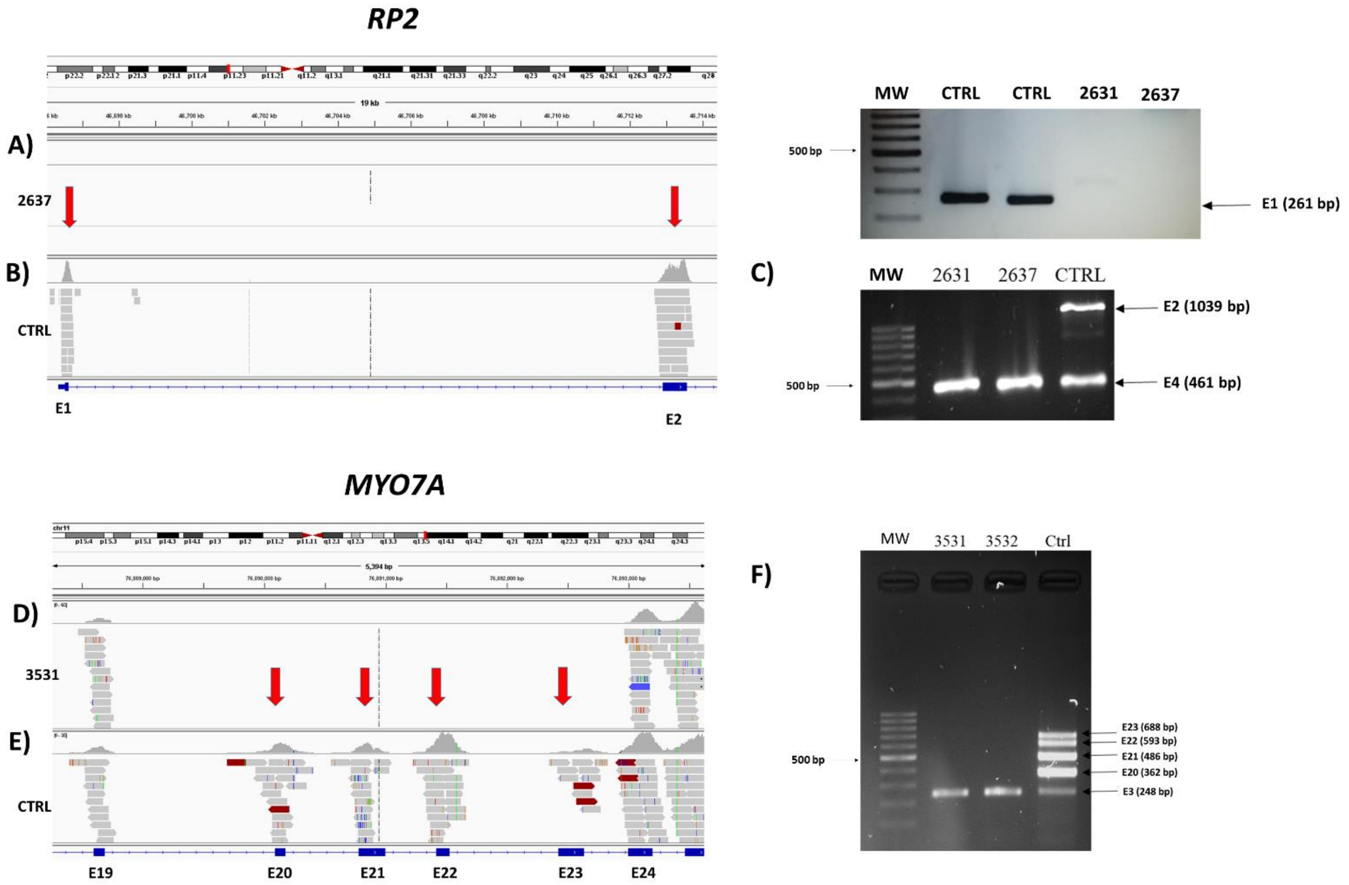

Supp. Figure S2.- Coverage analysis revealed large intragenic deletions in 2 different RD cases. Aligned reads in .bam files of patients 2637 **(A),** 3531 **(D),** diagnosed with AR RP and Usher syndrome, respectively, were visualized through IGV and compared to control samples **(B, E)** from the same NGS run. In **A**, a hemizygous deletion of RP2 exons 1-2 (E1 and E2) is demonstrated. In **D**, homozygous deletion of MYO7A exons 20-23 (E20-E23) is observed; red arrows indicate the exonic region(s) with no detectable reads. Subsequent PCR analysis of apparently deleted exons in DNA from the patients, affected relatives (2631 and 3532), and from control DNA **(C, F)** was used to confirm intragenic deletions detected through coverage analysis. **MW:** Molecular weight ladder (100 bp); **Ctrl:** Control DNA.

Supp. Table S1.- List of retinal dystrophy genes included in the ClearSeq Inherited Disease sequencing panel.

GENE	LOCUS	MIM#	INHERITANCE	NM_ID	RELATED CONDITIONS
Only Non-Syndromic RDs					
ABCA4	1p22.1	601691	AR	NM_000350.2	STGD; RP; MD; CRD
ADAM9	8p11.22	602713	AR	NM_003816.2	CRD
AIPL1	17p13.2	604392	AD, AR	NM_014336.3	LCA; RP; CRD
BEST1	11q12.3	607854	AD, AR	NM_001139443.1	MD; RP; vitreoretinochoroidopathy
PCARE	2p23.2	613425	AR	NM_001029883.2	RP
C1QTNF5	11q23.3	608752	AD	NM_015645.4	Late-onset retinal degeneration
CA4	17q23.1	114760	AD	NM_000717.3	RP
CABP4	11q13.2	608965	AR	NM_145200.3	CSNB; LCA
CACNA1F	Xp11.23	300110	XL	NM_005183.2	CSNB; CRD
CACNA2D4	12p13.33	608171	AR	NM_172364.4	CRD
CDHR1	10q23.1	609502	AR	NM_033100.3	CRD
CERKL	2q31.3	608381	AR	NM_001030311.2	RP; CRD
CHM	Xq21.2	300390	XL	NM_000390.2	Choroideremia
CNGA1	4p12	123825	AR	NM_001142564.1	RP
CNGA3	2q11.2	600053	AR	NM_001298.2	Achromatopsia; CRD
CNGB1	16q21	600724	AR	NM_001297.4	RP
CNGB3	8q21.3	605080	AR	NM_019098.4	Achromatopsia; CRD
CRB1	1q31.3	604210	AD; AR	NM_201253.2	LCA; RP; Pigmented paravenous chorioretinal atrophy
CRX	19q13.33	602225	AD; AR	NM_000554.4	LCA; RP
CYP4V2	4q35.1-q35.2	608614	AR	NM_207352.3	Bietti crystalline corneoretinal dystrophy
DHDDS	1p36.11	608172	AR	NM_024887.3	RP
EFEMP1	2p16.1	601548	AD	NM_001039348.2	Doyne honeycomb degeneration of retina
EYS	6q12	612424	AR	NM_001142800.1	RP
FAM161A	2p15	613596	AR	NM_001201543.1	RP
FBLN5	14q32.12	604580	AD	NM_006329.3	AMD
FSCN2	17q25.3	607643	AD	NM_001077182.2	RP, AMD
FZD4	11q14.2	604579	AD	NM_012193.3	Exudative vitreoretinopathy
GDF6	8q22.1	601147	AR	NM_001001557.2	LCA
GNAT1	3p21.31	139330	AD; AR	NM_000172.3	CSNB
GNAT2	1p13.3	139340	AR	NM_005272.3	Achromatopsia
GPR179	17q12	614515	AR	NM_001004334.2	CSNB
GRK1	13q34	180381	AR	NM_002929.2	CSNB; Oguchi disease
GRM6	5q35.3	604096	AR	NM_000843.3	CSNB
GUCA1A	6p21.1	600364	AD	NM_000409.3	CRD
GUCA1B	6p21.1	602275	AD	NM_002098.5	RP; MD
GUCY2D	17p13.1	600179	AD; AR	NM_000180.3	CRD; LCA
HK1	10q22.1	142600	AD	NM_033497.2	RP
IDH3B	20p13	604526	AR	NM_006899.3	RP
IMPDH1	7q32.1	146690	AD	NM_000883.3	LCA; RP
IMPG2	3q12.3	607056	AD; AR	NM_016247.3	RP; MD
KCNJ13	2q37.1	603208	AD; AR	NM_002242.4	Vitreoretinal degeneration; LCA
KCNV2	9p24.2	607604	AR	NM_133497.3	CRD
KLHL7	7p15.3	611119	AD	NM_001031710.2	RP
LCA5	6q14.1	611408	AR	NM_181714.3	LCA
LRAT	4q32.1	604863	AR	NM_004744.3	LCA; RP
MAK	6p24.2	154235	AR	NM_001242957.1	RP
MERTK	2q13	604705	AR	NM_006343.2	RP

NMNAT1	1p36.22	608700	AR	NM_022787.3	LCA
NR2E3	15q23	604485	AD; AR	NM_014249.3	RP; Enhanced S-cone syndrome
NRL	14q11-q12	162080	AD; AR	NM_006177.3	RP
NYX	Xp11.4	300278	XL	NM_022567.2	CSNB
OPN1LW	Xq28	300822	XL	NM_020061.4	Blue cone monochromacy; Colorblindness
OPN1MW	Xq28	300821	XL	NM_000513.2	Blue cone monochromacy; Colorblindness
OPN1SW	7q32.1	613522	AD	NM_001708.2	Colorblindness
PDE6A	5q32	180071	AR	NM_000440.2	RP
PDE6B	4p16.3	180072	AD; AR	NM_000283.3	CSNB; RP
PDE6C	10q23.33	600827	AR	NM_006204.3	CRD
PDE6G	17q25.3	180073	AR	NM_002602.3	RP
PDE6H	12p12.3	610024	AD; AR	NM_006205.2	CRD; Achromatopsia
PITPNM3	17p13.2p13.1	608921	AD	NM_031220.3	CRD
PLA2G5	1p36.13	601192	AR	NM_000929.2	Fleck retina
PRCD	17q25.1	610598	AR	NM_001077620.2	RP
PROM1	4p15.32	604365	AD; AR	NM_006017.2	CRD; MD; RP; STGD
PRPF3	1q21.2	607301	AD	NM_004698.2	RP
PRPF6	20q13.33	613979	AD	NM_012469.3	RP
PRPF8	17p13.3	607300	AD	NM_006445.3	RP
PRPF31	19q13.42	606419	AD	NM_015629.3	RP
PRPH2	6p21.1	179605	AD; AR	NM_000322.4	Choroidal dystrophy; LCA; MD; RP
RAX2	19p13.3	610362	AD	NM_032753.3	CRD; AMD
RB1	13q14.2	614041	AD	NM_000321.2	Retinoblastoma
RBP3	10q11.22	180290	AR	NM_002900.2	RP
RD3	1q32.3	180040	AR	NM_183059.2	LCA
RDH5	12q13.2	601617	AD; AR	NM_002905.3	CRD; Fundus albipunctatus
RDH12	14q24.1	608830	AR	NM_152443.2	LCA; RP
RGR	10q23.1	600342	AD; AR	NM_002921.3	Chorioretinal atrophy or degeneration; RP
RGS9	17q24.1	604067	AR	NM_003835.3	Delayed cone adaptation
RGS9BP	19q13.11	607814	AR	NM_207391.2	Delayed cone adaptation
RHO	3q22.1	180380	AD; AR	NM_000539.3	CSNB; RP; Retinitis punctata albescens
RIMS1	6q13	606629	AD	NM_014989.5	CRD
RLBP1	15q26.1	180090	AD; AR	NM_000326.4	Bothnia retinal dystrophy; Fundus albipunctatus; RP; Retinitis punctata albescens
ROM1	11q12.3	180721	AD; AR	NM_000327.3	RP
RP1	8q11.2-q12.1	603937	AD; AR	NM_006269.1	RP
RP2	Xp11.3	312600	XL	NM_006915.2	RP
RP9	7p14.3	607331	AD	NM_203288.1	RP
RP1L1	8p23.1	608581	AD; AR	NM_178857.5	Occult macular dystrophy; RP
RPE65	1p31.3	180069	AR	NM_000329.2	LCA; RP
RPGR	Xp11.4	312610	XL	NM_001034853.1	CRD; MD; RP
RPGRIP1	14q11.2	605446	AR	NM_020366.3	CRD; LCA
RS1	Xp22.13	312700	XL	NM_000330.3	Retinoschisis
SAG	2q37.1	181031	AR	NM_000541.4	RP; Oguchi disease
SEMA4A	1q22	607292	AD; AR	NM_001193301.1	CRD; RP
SLC24A1	15q22.31	603617	AR	NM_004727.2	CSNB
SNRNP200	2q11.2	601664	AD	NM_014014.4	RP
SPATA7	14q31.3	609868	AR	NM_018418.4	LCA; RP
TEAD1	11p15.3	189967	AD	NM_021961.5	Chorioretinal atrophy
TIMP3	22q12.3	188826	AD	NM_000362.4	MD

TMEM126A	11q14.1	612988	AR	NM_032273.3	Optic Atrophy
TOPORS	9p21.1	609507	AD	NM_005802.4	RP
TRPM1	15q13.3	603576	AR	NM_001252020.1	CSNB
TSPAN12	7q31.31	613138	AD	NM_012338.3	Exudative vitreoretinopathy
TULP1	6p21.31	602280	AR	NM_003322.3	LCA; RP
UNC119	17q11.2	604011	AD	NM_005148.3	CRD
VCAN	5q14.2-q14.3	118661	AD	NM_004385.4	Wagner syndrome
ZNF513	2p23.3	613598	AR	NM_144631.5	RP
Syndromic and Non-Syndromic RDs					
ALMS1	2p13.1	606844	AR	NM_015120.4	Alström syndrome; NS CRD
ARL6	3q11.2	608845	AR	NM_032146.4	Bardet-Biedl syndrome; NS RP
BBS1	11q13.2	209901	AR	NM_024649.4	Bardet-Biedl syndrome; NS RP
BBS2	16q13	606151	AR	NM_031885.3	Bardet-Biedl syndrome; NS RP
C8orf37	8q22.1	614477	AR	NM_177965.3	Bardet-Biedl syndrome; NS RP; CRD
CEP290	12q21.32	610142	AR	NM_025114.3	Bardet-Biedl syndrome; Joubert syndrome; NS LCA; Meckel syndrome; Senior-Loken syndrome
CLN3	16p12.1	607042	AR	NM_001042432.1	Ceroid lipofuscinosis; NS RP
ELOVL4	6q14.1	605512	AD	NM_022726.3	STGD; Spinocerebellar ataxia
CLRN1	3q25.1	606397	AR	NM_001195794.1	Usher syndrome; NS RP
HGSNAT	8p11.2-p11.1	610453	AR	NM_152419.2	Mucopolysaccharidosis; NS RP
IFT140	16p13.3	614620	AR	NM_014714.3	Short-rib thoracic dysplasia with or without polydactyly; NS RP
IQCB1	3q13.33	609237	AR	NM_001023570.2	Senior-Loken syndrome; NS LCA
LRP5	11q13.2	603506	AD; AR	NM_002335.2	NS Exudative vitreoretinopathy; Osteoporosis-pseudoglioma syndrome
MFN2	1p36.22	608507	AD; AR	NM_014874.3	NS Optic Atrophy; Charcot-Marie-Tooth disease
MFSD8	4q28.2	611124	AR	NM_152778.2	Ceroid lipofuscinosis; NS MD
MVK	12q24.11	251170	AR	NM_000431.2	Hyper-IgD syndrome; NS RP
NDP	Xp11.3	300658	XL	NM_000266.3	NS Exudative vitreoretinopathy; Norrie disease
NEUROD1	2q31.3	601724	AR	NM_002500.4	Early onset diabetes, neurologic abnormalities and retinal degeneration; NS RP
OFD1	Xp22.2	300170	XL	NM_003611.2	Joubert syndrome; Orofaciodigital syndrome; NS RP
OPA1	3q29	605290	AD; AR	NM_130837.2	NS Optic atrophy; Behr syndrome
POMGNT1	1p34.1	606822	AR	NM_001243766.1	Muscular dystrophy-dystroglycanopathy; NS RP
TTC8	14q31.3	608132	AR	NM_144596.2	Bardet-Biedl syndrome; NS RP
USH2A	1q41	608400	AR	NM_206933.2	Usher syndrome; NS RP
Only Syndromic RDs					
ABCC6	16p13.11	603234	AD, AR	NM_001171.5	Pseudoxanthoma elasticum
ABHD12	20p11.21	613599	AR	NM_015600.4	PHARC; Usher Syndrome
ADAMTS18	16q23.1	607512	AR	NM_199355.2	Knobloch syndrome
ADGRV1 *~~GPR98~~*	5q14.3	602851	AD, AR	NM_032119.3	Usher Syndrome; Febrile Convulsions
AHI1	6q23.3	608894	AR	NM_017651.4	Joubert syndrome
ATXN7	3p14.1	607640	AD	NM_001177387.1	Spinocerebellar ataxia
BBS4	15q24.1	600374	AR	NM_033028.4	Bardet-Biedl syndrome
BBS5	2q31.1	603650	AR	NM_152384.2	Bardet-Biedl syndrome
BBS7	4q27	607590	AR	NM_176824.2	Bardet-Biedl syndrome
BBS9	7p14.3	615986	AR	NM_198428.2	Bardet-Biedl syndrome

BBS10	12q21.2	610148	AR	NM_024685.3	Bardet-Biedl syndrome
BBS12	4q27	610683	AR	NM_152618.2	Bardet-Biedl syndrome
C12orf65	12q24.31	613541	AR	NM_152269.4	Spastic paraplegia
CC2D2A	4p15.32	612013	AR	NM_001080522.2	Joubert syndrome ; COACH syndrome; Meckel syndrome
CDH3	16q22.1	114021	AR	NM_001793.4	Syndromic MD
CDH23	10q22.1	605516	AR	NM_022124.5	Usher Syndrome
CIB2	15q25.1	605564	AR	NM_006383.3	Usher syndrome
CNNM4	2q11.2	607805	AR	NM_020184.3	Jalili syndrome
COL11A1	1p21.1	120280	AD	NM_080629.2	Stickler syndrome; Marshall syndrome
COL2A1	12q13.11	120140	AD	NM_001844.4	Stickler syndrome; Bone dysplasias; Developmental disorders; Osteoarthritic diseases, Syndromic disorders
COL9A1	6q13	120210	AD; AR	NM_001851.4	Stickler syndrome; Multiple epiphyseal dysplasia
DFNB31/ WHRN	9q32	607928	AR	NM_015404.3	Usher syndrome
DMD	Xp21.2-p21.1	300377	XL	NM_004006.2	Duchenne muscular dystrophy
ERCC6	10q11.23	609413	AR	NM_000124.3	Cockayne syndrome; AMD
FLVCR1	1q32.3	609144	AR	NM_014053.3	Ataxia, posterior column with RP
GNPTG	16p13.3	607838	AR	NM_032520.4	RP and skeletal abnormalities
HARS	5q31.3	142810	AR	NM_002109.4	Usher syndrome
HMX1	4p16.1	142992	AR	NM_018942.2	Oculoauricular syndrome
INVS	9q31.1	243305	AR	NM_014425.3	Senior-Loken syndrome; Nephronophthisis
ITM2B	13q14.2	603904	AD	NM_021999.4	Retinal dystrophy; Dementia
JAG1	20p12.2	601920	AD	NM_000214.2	Alagille syndrome
KIF11	10q23.33	148760	AD	NM_004523.3	Microcephaly, lymphedema and chorioretinopathy
LAMA1	18p11.31	150320	AR	NM_005559.3	Poretti-Boltshauser syndrome
LZTFL1	3p21.31	606568	AR	NM_020347.3	Bardet-Biedl syndrome
MFRP	11q23.3	606227	AR	NM_031433.3	Microphthalmos and retinal disease syndrome
MKKS	20p12.2	604896	AR	NM_170784.2	Bardet-Biedl syndrome
MKS1	17q22	609883	AR	NM_017777.3	Bardet-Biedl syndrome; Joubert syndrome; Meckel syndrome
MYO7A	11q13.5	276903	AR	NM_000260.3	Usher syndrome
NBAS	2p24.3	608025	AR	NM_015909.3	Short stature, optic nerve atrophy, and Pelger-Huet anomaly
NPHP1	2q13	607100	AR	NM_000272.3	Joubert syndrome; Nephronophthisis; Senior-Loken syndrome
NPHP3	3q22.1	608002	AR	NM_153240.4	Senior-Loken syndrome; Nephronophthisis
NPHP4	1p36.31	607215	AR	NM_015102.3	Senior-Loken syndrome; Nephronophthisis
OAT	10q26.13	613349	AR	NM_000274.3	Gyrate atrophy of choroid and retina with or without ornithinemia
OPA3	19q13.32	606580	AD; AR	NM_001017989.2	3-methylglutaconic aciduria; Optic atrophy and cataract
OTX2	14q22.3	600037	AD	NM_021728.3	Retinal dystrophy, early-onset, with or without pituitary dysfunction; Syndromic microphthalmia
PANK2	20p13	606157	AR	NM_153638.2	HARP syndrome

PAX2	10q24.31	167409	AD	NM_003990.3	Papillorenal syndrome
PCDH15	10q21.1	605514	AR	NM_001142763.1	Usher syndrome
PDZD7	10q24.31	605472	AR; Digenic	NM_001195263.1	Usher syndrome
PEX1	7q21.2	602136	AR	NM_000466.2	Refsum disease
PEX2	8q21.13	170993	AR	NM_001172086.1	Refsum disease
PEX7	6q23.3	601757	AR	NM_000288.3	Refsum disease
PGK1	Xq21.1	300653	XL	NM_000291.3	RP with myopathy
PHYH	10p13	602026	AR	NM_006214.3	Refsum disease
PNPLA6	19p13.2	603197	AR	NM_001166111.1	Laurence-Moon syndrome; Boucher-Neuhauser syndrome; Oliver-McFarlane syndrome
PRPS1	Xq22.3	311850	XL	NM_002764.3	Arts syndrome; Charcot-Marie-Tooth disease
RBP4	10q23.33	180250	AR	NM_006744.3	Retinal dystrophy, iris coloboma, and comedogenic acne syndrome
RPGRIP1L	16q12.2	610937	AR	NM_015272.2	COACH syndrome; Joubert syndrome
SDCCAG8	1q43-q44	613524	AR	NM_006642.3	Bardet-Biedl syndrome; Senior-Loken syndrome
TIMM8A	Xq22.1	300356	XL	NM_004085.3	Mohr-Tranebjaerg syndrome
TMEM216	11q12.2	613277	AR	NM_001173991.2	Joubert syndrome
TMEM237	2q33.1	614423	AR	NM_001044385.2	Joubert syndrome
TREX1	3p21.31	606609	AD	NM_016381.4	Vasculopathy, retinal, with cerebral leukodystrophy
TRIM32	9q33.1	602290	AR	NM_012210.3	Bardet-Biedl syndrome
TTPA	8q12.3	600415	AD; AR	NM_000370.3	RP and/or ataxia
TUBGCP6	22q13.33	610053	AR	NM_020461.3	Microcephaly and chorioretinopathy
USH1C	11p15.1	605242	AR	NM_153676.3	Usher syndrome
USH1G	17q25.1	607696	AR	NM_173477.2	Usher syndrome
VPS13B	8q22.2	607817	AR	NM_017890.4	Cohen syndrome
WDPCP	2p15	613580	AR	NM_015910.5	Bardet-Biedl syndrome
WDR19	4p14	608151	AR	NM_025132.3	Cranioectodermal dysplasia; Short-rib thoracic dysplasia; Nephronophthisis; Senior-Loken syndrome
WFS1	4p16.1	606201	AD; AR	NM_006005.3	Wolfram syndrome

The panel used in this study contains 199 RD-related genes, including 104 genes implicated only in non-syndromic RDs, 23 genes involved in both syndromic and non-syndromic forms of RDs, and 72 genes implicated only in syndromic RDs. Gene information was obtained from the Retinal Information Network (RETNET) and the Online Mendelian Inheritance in Man (OMIM) catalog (Accessed december 2018). NM_ID represents the transcript used for variant analysis and sequencing data analysis. AMD: Age-related macular degeneration; CRD: Cone-Rod dystrophy; CSNB: Congenital stationary night blindness; LCA: Leber Congenital Amaurosis; MD: Macular dystrophy; RP: Retinitis Pigmentosa; STGD: Stargardt disease; NS: Non-syndromic; AD: Autosomal Dominant; AR: Autosomal Recessive; XL: X-Linked.

Supp. Table S2.- American College of Medical Genetics and Genomics (ACMG) criteria for classification of novel variants as pathogenic or likely pathogenic.

Patient	Gene	c.DNA variant	Protein alteration	ACMG criteria
3632	ABCA4	c.1417_1420dupATTA	p.Thr474AsnfsTer4	Pathogenic (PVS1, PM3, PP1,PP4)
3585		c.4854G>C	p.Trp1618Cys	Likely Pathogenic (PM2, PM3, PP3, PP4)
1175		c.6282+3A>T	splicing	Likely pathogenic (PM2, PM3, PP3, PP4)
3286		c.3383A>G	p.Asp1128Gly	Likely pathogenic (PM2,PM3, PP3, PP4)
3286		c.4804delA	p.Ile1602TyrfsTer8	Pathogenic (PVS1, PM2, PP4)
3602	AHI1	c.2029A>C	p.Thr677Pro	Likely pathogenic (PM1, PM2, PP1, PP3, PP4)
1521	ARL6	c.373dupA	p.Ile125AsnfsTer7	Pathogenic (PVS1, PM2, PP4)
2831	BBS9	c.1329+1738C>T	Deep-intronic	Likely pathogenic (PS3, PM2, PP3, PP4)
1977	CDHR1	c.963G>C	p.Gln321His	Likely pathogenic (PM2, PM3, PP1, PP3, PP4)
1977		c.2041-2A>C	splicing	Pathogenic (PVS1, PM2, PP1, PP3, PP4)
3593	CEP290	c.2605C>T	p.Gln869Ter	Pathogenic (PVS1, PM2, PP4)
2792	CERKL	c.1633_1636dupATCA	p.Ser546AsnfsTer21	Pathogenic (PVS1, PM2, PP1, PP4)
2699		c.424_427delAATT	p.Asn142Ter	Pathogenic (PVS1, PM2, PM3, PP4)
2699		c.1030_1039dupTGGGTTCT	p.Ser347LeufsTer77	Pathogenic (PVS1, PM2, PM3, PP4)
3919	CLN3	c.266G>A	p.Arg89Gln	Pathogenic (PS1, PM2, PP1, PP2, PP3, PP4)
3436	CLRN1	c.41G>A	p.Gly14Glu	Likely Pathogenic (PM2, PM3, PM5, PP3, PP4)
3480	CRB1	c.2797T>C	p.Cys933Arg	Likely Pathogenic (PM1, PM2, PM3, PP3, PP4)
197		c.3158T>A	p.Met1053Lys	Likely Pathogenic (PM2, PM3, PM5, PP3, PP4)
3043		c.3822C>A	p.Cys1274Ter	Pathogenic (PVS1, PM2, PP4)
3662	GNAT1	c.282delT	p.Ala95HisfsTer9	Pathogenic (PVS1, PM2, PP4)
2996	GUCA1A	c.328_337delGATGAGCTGC	p.Asp110SerfsTer18	Pathogenic (PVS1, PM2, PP4)
2257	GUCY2D	c.1157delA	p.Gln386ArgfsTer9	Pathogenic (PVS1, PM2, PP4)
1985		c.982G>C	p.Ala328Pro	Likely pathogenic (PM2, PM3, PP1, PP4)
1985		c.997G>A	p.Glu333Lys	Likely Pathogenic (PM2, PM3, PP1, PP3, PP4)
3525		c.2705T>C	p.Val902Ala	Likely pathogenic (PM1, PM2, PP1, PP3, PP4)
3868	IDH3B	c.857G>A	p.Gly286Glu	Likely pathogenic (PM1, PM2 ,PP2, PP3, PP4)
1830	IFT140	c.2786delC	p.Thr929SerfsTer21	Pathogenic (PVS1, PM2, PM3, PP4)
3068		c.4252C>T	p.Gln1418Ter	Pathogenic (PVS1, PM2, PP1, PP4)
3332	IMPG2	c.3093_3097dupTGGAG	p.Glu1033ValfsTer13	Pathogenic (PVS1, PM2, PM3, PP1, PP4)
3332		c.2038delG	p.Glu680SerfsTer21	Pathogenic (PVS1, PM2, PM3, PP1, PP4)
1602		c.2887A>G	p.Ser963Gly	Likely pathogenic (PM1, PM2, PP1, PP3, PP4)
1274	LRAT	c.614_615delCT	p.Ser205TyrfsTer51	Pathogenic (PVS1, PM2, PP1,PP4)
1140	MERTK	c.2531G>A	p.Arg844His	Pathogenic (PS*, PM1, PM2, PM5, PP3, PP4)
3531	MYO7A	c.(2282+1_2283-1_(2904+1_2905-1)del (exon 20-23 deletion)	-----	Pathogenic (PVS1, PM2, PP1, PP4)
3635	PDE6A	c.2302G>T	p.Glu768Ter	Pathogenic (PVS1, PM2, PP1, PP4)
3527	RP1	c.3150delA	p.Lys1050AsnfsTer7	Pathogenic (PVS1, PM2, PP4)
2637	RP2	Del exon 2	c.(?_-1)_(768+1_769-1)del	Pathogenic (PVS1, PM2, PP1, PP4)
3354		c.969+2T>G	splicing	Pathogenic (PVS1, PM2, PP1, PP4)
3533		c.1A>G	p.Met1?	Pathogenic (PVS1, PS*, PM2, PP4)
3544	RPE65	c.405T>A	p.Asn135Lys	Likely pathogenic (PM2, PP1, PP2, PP3, PP4)
3751		c.61delG	p.Glu21Asnfster10	Pathogenic (PVS1, PM2, PM3, PP2, PP4)
3261		c.386C>T	p.Thr129Ile	Likely pathogenic (PM2, PM3,PP2, PP3, PP4)
2934		c.190delC	p.Gln64LysfsTer30	Pathogenic (PVS1, PM2, PM3, PP4)
3340	RPGR	c.1859_1860delAG	p.Lys620ArgfsTer9	Pathogenic (PVS1, PM2, PP4)
3483	RPGRIP1	c.1624delG	p.Ala542GlnfsTer2	Pathogenic (PVS1, PM2, PP4)
3592		c.1116delA	p.Lys372AsnfsTer3	Pathogenic (PVS1, PM2, PP4)
2583	TTC8	c.674G>A	p.Trp225Ter	Pathogenic (PVS1, PM2, PP4)
2669	TULP1	c.1102G>A	p.Gly368Arg	Likely pathogenic (PM2, PM5,PP1, PP3, PP4)
2666	USH2A	c.11387C>T	p.Pro3796Leu	Likely pathogenic (PM1, PM2, PP1, PP3, PP4)
1180		c.5218delA	p.Ile1740PhefsTer10	Pathogenic (PVS1, PM1, PM2, PM3, PP1, PP4)

3268		c.3629T>C	p.Leu1210Pro	Likely pathogenic (PM1, PM2, PM3, PP3, PP4)
3776		c.11389+1G>A	Splicing	Pathogenic (PVS1, PS*, PM2, PP4)
4066		c.13448C>T	p.Pro4450Ser	Likely pathogenic (PM1, PM2, PM3, PP3, PP4)

ACMG criteria:

Very strong (PVS1): null variant (nonsense, frameshift, canonical ±1 or 2 splice sites, initiation codon, single or multiexon deletion) in a gene where LOF is a known mechanism of disease.

Strong (PS): 1. Same amino acid change as a previously established pathogenic variant regardless of nucleotide change

2. De novo (both maternity and paternity confirmed) in a patient with the disease and no family history

*Variant segregates in multiple affected family members

3.Moderate (PM): 2. Absent from controls (or at extremely low frequency if recessive) in Exome Sequencing Project, 1000 Genomes Project, or Exome Aggregation Consortium.

3. For recessive disorders, detected in trans with a pathogenic variant

5. Novel missense change at an amino acid residue where a different missense change determined to be pathogenic has been seen before.

6. Assumed de novo, but without confirmation of paternity and maternity.

Supporting (PP): 1. Cosegregation with disease in multiple affected family members in a gene definitively known to cause the disease.

3. Multiple lines of computational evidence support a deleterious effect on the gene or gene product (conservation, evolutionary, splicing impact, etc.).

4. Patient’s phenotype or family history is highly specific for a disease with a single genetic etiology

From: Richards, S., Aziz, N., Bale, S., Bick, D., Das, S., Gastier-Foster, J., … & Rehm, H.L. ACMG Laboratory Quality Assurance Committee. (2015). Standards and guidelines for the interpretation of sequence variants: a joint consensus recommendation of the American College of Medical Genetics and Genomics and the Association for Molecular Pathology. *Genetics in Medicine*, 17:405-24

Supp. Table S3: *In silico* pathogenicity analysis and population frequency of novel missense and splicing variants identified in our study.

ID	Gene	NM ID	cDNA Change	Protein change	SIFT	POLYPHEN-2	MUTATION TASTER 2	ALAMUT	GnomAD
3585	*ABCA4*	NM_000350.2	c.4854G>C	p.Trp1618Cys	deleterious (0)	probably damaging (1)	Disease Causing (1)	N/A	0.00001593
3286	*ABCA4*	NM_000350.2	c.3383A>G	p.Asp1128Gly	deleterious (0)	possibly damaging (0.894)	Disease Causing (1)	N/A	N/A
1175	*ABCA4*	NM_000350.2	c.6282+3A>T	N/A	N/A	N/A	N/A	Prevents donor site (4/5)	N/A
3602	*AHI1*	NM_017651.4	c.2029 A>C	p. Thr677Pro	deleterious (0)	probably damaging (1)	Disease Causing (1)	N/A	N/A
2831	*BBS9*	NM_198428.2	c.1329+1738C>T	N/A	N/A	N/A	N/A	Creates new donor site (4/5)	N/A
1977	*CDHR1*	NM_033100.3	c.963G>C	p.Gln321His	deleterious (0)	probably damaging (0.978)	Disease Causing (1)	N/A	0.000003985
			c.2041-2A>C	N/A	N/A	N/A	N/A	Prevents acceptor site (5/5)	0.00002394
3919	*CLN3*	NM_001042432.1	c.266G>A	p.Arg89Gln	tolerated (0.37)	probably damaging (0.997)	Disease Causing (0.999)	N/A	N/A
3436	*CLRN1*	NM_001195794.1	c.41 G>A	p.Gly14Glu	deleterious (0.01)	probably damaging (1)	Disease Causing (1)	N/A	N/A
3480	*CRB1*	NM_201253.2	c.2797T>C	p.Cys933Arg	deleterious (0)	probably damaging (0.998)	Disease Causing (1)	N/A	N/A
197	*CRB1*	NM_201253.2	c.3158T>A	p.Met1053Lys	deleterious (0)	probably damaging (0.994)	Disease Causing (1)	N/A	N/A
3525	*GUCY2D*	NM_000180.3	c.2705T>C	p.Val902Ala	deleterious (0)	probably damaging (1)	Disease Causing (1)	N/A	N/A
1985	*GUCY2D*	NM_000180.3	c.982G>C	p.Ala328Pro	deleterious (0)	probably damaging (1)	Disease Causing (9.27)	N/A	N/A
			c.997G>A	p.Glu333Lys	deleterious (0)	probably damaging (0.983)	Disease Causing (0.999)	N/A	0.000004255
1602	*IMPG2*	NM_016247.3	c.2887 A>G	p.Ser963Gly	deleterious (0)	probably damaging (1)	Disease Causing (1)	N/A	N/A
1140	*MERTK*	NM_006343.2	c.2531G>A	p.Arg844His	deleterious (0.02)	probably damaging (1)	Disease Causing (1)	N/A	0.00001193
3354	*RP2*	NM_006915.2	c.969+2T>G	N/A	N/A	N/A	N/A	Prevents donor site (4/4)	N/A
3261	*RPE65*	NM_000329.2	c.386 C>T	p.Thr129Ile	tolerated (0.14)	probably damaging (1)	Disease Causing (1)	N/A	0.000003980
3544	*RPE65*	NM_000329.2	c.405T>A	p.Asn135Lys	tolerated (0.28)	probably damaging (0.999)	Disease Causing (1)	N/A	N/A
2666	*USH2A*	NM_206933.2	c.11387C>T	p.Pro3796Leu	deleterious (0)	probably damaging (0.999)	Disease Causing (1)	N/A	0.00001621
3268	*USH2A*	NM_206933.2	c.3629T>C	p.Leu1210Pro	deleterious (0)	probably damaging (1)	Disease Causing (1)	N/A	N/A
3776	*USH2A*	NM_206933.2	c.11389+1G>A	N/A	N/A	N/A	N/A	Prevents donor site (4/4)	N/A
4066	*USH2A*	NM_206933.2	c.13348 C>T	p.Pro4450Ser	tolerated (0.15)	probably damaging (1)	Disease Causing (1)	N/A	0.0000279
2669	*TULP1*	NM_003322.3	c.1102G>A	p.Gly368Arg	deleterious (0.01)	probably damaging (1)	Disease Causing (1)	N/A	0.000003979

Missense and splicing site predictions were generated using the Alamut Visual v.2.10 (https://www.interactive-biosoftware.com/alamut-visual/). This program uses the Sorting Intolerant from Tolerant (SIFT, https://sift.bii.a-star.edu.sg/), Polymorphism Phenotyping v2 (PolyPhen-2, http://genetics.bwh.harvard.edu/pph2/), and mutation Taster 2 (http://www.mutationtaster.org/) algorithms to make pathogenicity predictions of missense variants (default parameters and program options were used). Alamut also uses 5 distinct algorithms (SpliceSiteFinder-like, MaxEntScan, NNPLICE, GeneSplicer, and Human Splicing Finder) to evaluate effects of variants on the splicing process. GnomAD database was used to obtain population frequencies. "()" next to SIFT and PolyPhen-2 columns indicate Pathogenicity Scores; "()" next to the Mutation Taster column indicate p-Values; "()" next to Alamut column indicates the number of splicing site algorithms used by Alamut that reached the same prediction. N/A: Not available. ***In silico* tools ranges and scores**: SIFT ranges: The amino acid substitution is predicted damaging if the score is <= 0.05, and tolerated if the score is > 0.05. **Polyphen-2**: 0.0 to 0.15: Variant is predicted to be benign; 0.15 to 1.0: Variants are possibly damaging; 0.85 to 1.0: Variant is predicted to be damaging. **Mutation Taster 2**: This tool provides a probability value of the prediction; a value close to 1 indicates a high "security" of the prediction; scores below 0.5 indicate a low confidence prediction.

Supp. Table S4: *In silico* pathogenicity predictions and population frequencies for heterozygous variants detected in recessive genes from unsolved cases.

ID	Gene	Genotype	cDNA Change	Protein change	SIFT	PolyPhen-2	Mutation Taster 2	Splicing	GnomAD	Refs.
Retinitis Pigmentosa (AR)										
	CNGB1	Heterozygous	c.458+8C>T	N/A	N/A	N/A	N/A	Creates acceptor site (2/5)	0.00005613	N/A
EC02	USH2A	Heterozygous	c.10999A>C	p.Thr3667Pro	deleterious (0.03)	possibly damaging (0.949)	polymorphism (1)	N/A	0.0000938	1
257	AHI1	Heterozygous	c.1585delA	p.Thr529HisfsTer4	N/A	N/A	N/A	N/A	N/A	N/A
		Heterozygous	c.3250A>C	p.Thr1084Pro	Tolerated (0.26)	Benign (0.001)	polymorphism (1)	N/A	0.000154	2
		Heterozygous	c.3443+1G>T	N/A	N/A	N/A	N/A	Prevents Donor Site (5/5)	0.0000619	2, 3
3349	EYS	Heterozygous	c.4402 G>C	p.Asp1468His	Deleterious (0)	probably damaging (0.996)	polymorphism (1)	N/A	0.000189	2, 3
Simplex RP										
EC01	CNGB1	Heterozygous	c.2656G>A	p.Ala886Thr	deleterious (0)	probably damaging (1)	disease causing (1)	N/A	0.00009361	N/A
	HGSNAT	Heterozygous	c.852G>T	p.Trp284Cys	tolerated (0.1)	benign (0.289)	disease causing (1)	N/A	0.000008136	N/A
EC03	NMNAT1	Heterozygous	c.769G>A	p.Glu257Lys	tolerated (0.25)	benign (0.089)	disease causing (1)	N/A	0.0006968	4
EC06	PDZD7	Heterozygous	c.2089delG	p.Ala697ProfsTer26	N/A	N/A	N/A	N/A	0.0002736	N/A
3362	USH2A	Heterozygous	c.13519T>C	p.Tyr4507His	deleterious (0)	probably damaging (1)	disease causing (1)	N/A	0.000004069	N/A
	LRAT	Heterozygous	c.658A>C	p.Ile220Leu	tolerated (0.31)	benign (0.004)	disease causing (0.976)	N/A	N/A	N/A
	CNGB3	Heterozygous	c.397C>T	p.His133Tyr	deleterious (0.03)	possibly damaging (0.885)	polymorphism (1)	N/A	0.000004061	N/A
3580	RD3	Heterozygous	c.541C>G	p.Leu181Val	tolerated (0.16)	benign (0.039)	polymorphism (1)	N/A	N/A	N/A
3865	PDE6B	Heterozygous	c.2206G>A	p.Val736Met	deleterious (0.01)	probably damaging (1)	disease causing (1)	N/A	0.0000122	N/A
Leber Congenital Amaurosis										
EC04	CRB1	Heterozygous	c.2290C>T	p.Arg764Cys	tolerated (0.2)	benign (0.007)	disease causing (0)	N/A	0.00007953	5
3337	PNPLA6	Heterozygous	c.3530G>A	p.Trp1177Ter	N/A	N/A	N/A	N/A	N/A	N/A
**Recessive Syndromic cases**										
	BBS2	Heterozygous	c.143G>A	p.Arg48Gln	tolerated (0.37)	possibly damaging (0.997)	disease causing (1)	N/A	0.000008127	N/A
2007	*IFT140*	Heterozygous	c.3502G>A	p.Glu1168Lys	deleterious (0.03)	possibly damaging (0.986)	disease causing (1)	N/A	0.00001828	N/A
	ADGRV1	Heterozygous	c.5944dupT	p.Ser1982PhefsTer2	N/A	N/A	N/A	N/A	N/A	N/A
3853	*USH2A*	Heterozygous	c.2276G>T	p.Cys759Phe	deleterious (0)	probably damaging (1)	disease Causing (1)	N/A	0.0009692	6
3573	*USH2A*	Heterozygous	c.2299delG	p.Glu767SerfsTer21	N/A	N/A	N/A	N/A	0.0007017	7
3470	*KCNV2*	Heterozygous	c.8_11delAACA	p.Lys3ArgfsTer96	N/A	N/A	N/A	N/A	0.00004692	8
**Simplex syndromic cases**										
EC08	*USH2A*	Heterozygous	c.1850G>A	p.Cys617Tyr	deleterious (0)	probably damaging (1)	disease Causing (1)	N/A	N/A	N/A
3292	*RPGRIP1*	Heterozygous	c.2775G>A	p.Trp925Ter	N/A	N/A	N/A	N/A	0.000008123	N/A

All missense and splicing site predictions were generated using the Alamut Visual v.2.10 (https://www.interactive-biosoftware.com/alamut-visual/). This program uses the Sorting Intolerant from Tolerant (SIFT, https://sift.bii.a-star.edu.sg/), Polymorphism Phenotyping v2 (PolyPhen-2, http://genetics.bwh.harvard.edu/pph2/), and mutation Taster 2 (http://www.mutationtaster.org/) algorithms to make pathogenicity predictions of missense variants (default parameters and program options were used). The GnomAD database was used to obtain population frequencies. “()” next

to SIFT and PolyPhen-2 columns indicate Pathogenicity Scores; “()” next to the Mutation Taster column indicate p-Values; “()” next to the Splicing column indicates the number of splicing site algorithms used in Alamut that reached the same prediction. N/A: Not available. ***In silico* tools ranges and scores**: **SIFT** ranges: The amino acid substitution is predicted damaging if the score is <= 0.05, and tolerated if the score is > 0.05. **Polyphen-2**: 0.0 to 0.15: Variant is predicted to be benign; 0.15 to 1.0: Variants are possibly damaging; 0.85 to 1.0: Variant is predicted to be damaging. **Mutation Taster** 2: This tool provides a probability value of the prediction; a value close to 1 indicates a high “security” of the prediction; scores below 0.5 indicate a low confidence prediction.

Table S4 References

1. Miyagawa, M., Naito, T., Nishio, S. Y., Kamatani, N., Usami, S. (2013). Targeted exon sequencing successfully discovers rare causative genes and clarifies the molecular epidemiology of Japanese deafness patients. *PLoS One*, 8, e71381.

2. Wang J, Zhang VW, Feng Y, et al. Dependable and efficient clinical utility of target capture-based deep sequencing in molecular diagnosis of retinitis pigmentosa.Invest Ophthalmol Vis Sci. 2014;55:6213–6223. DOI:10.1167/iovs.14-14936

3. Ge, Z. et al., 2015. NGS-based Molecular diagnosis of 105 eyeGENE ® probands with Retinitis Pigmentosa. Scientific Reports, 5, pp.1–9.

4. Perrault, I., Hanein, S., Zanlonghi, X., Serre, V., Nicouleau, M., Defoort-Delhemmes, S., … Rozet, J. M. (2012). Mutations in NMNAT1 cause Leber congenital amaurosis with early-onset severe macular and optic atrophy. *Nature Genetics*, 44, 975-977

5. den Hollander, A. I., ten Brink, J. B., de Kok, Y. J, van Soest, S,, van den Born, L. I., van Driel, M. A., … Bergen, A. A. (1999). Mutations in a human homologue of Drosophila crumbs cause retinitis pigmentosa (RP12). *Nature Genetics*, 23, 217-221.

6. Rivolta, C., Sweklo, E. A., Berson, E. L., & Dryja, T. P. (2000). Missense mutation in the USH2A gene: association with recessive retinitis pigmentosa without hearing loss. *American Journal of Human Genetics*, 66, 1975-1978.

7. Eudy, J. D., Weston, M. D., Yao, S., Hoover, D. M., Rehm, H. L., Ma-Edmonds, M., …Sumegi, J. (1998). Mutation of a gene encoding a protein with extracellular matrix motifs in Usher syndrome type IIa. *Science*, 280, 1753-1757.

8. Wissinger, B., Schaich, S., Baumann, B., Bonin, M., Jägle, H., Friedburg, C., … Kohl S Large deletions of the KCNV2 gene are common in patients with cone dystrophy with supernormal rod response. *Human Mutation*, 32, 1398-1406.

Supp. Table S5.- Variants of Uncertain Significance identified in inconclusive cases.

**Case ID**	**1577**		**3537**		**37**		**3335**
**Gene**	***ALMS1***		***IFT140***		***RDH5***		***RP9***
**NM_ID**	NM_015120.4		NM_014714.3		NM_002905.3		NM_203288.1
**cDNA Change**	c.716C>A	c.9112T>C	c.3193G>A	c.2067+5G>A	c.797G>A	c.848C>G	c.44C>T
**Protein change**	p.Ala239Glu	p.Ser3038Pro	p.Glu1065Lys	N/A	p.Arg266Gln	p.Pro283Arg	p.Ala15Val
**SIFT**	tolerated (1)	deleterious (0)	tolerated (0.553)	N/A	tolerated (0.59)	tolerated (0.08)	tolerated (0.59)
**POLYPHEN-2**	benign (0)	probably damaging (0.998)	benign (0.1.20)	N/A	benign (0.029)	probably damaging (0.983)	probably damaging (0.998)
**MUTATION TASTER**	Polymorphism (1)	Polymorphism (0.985)	Disease Causing (0.999)	N/A	Polymorphism (1)	Disease Causing (0.999)	Polymorphism (0.98)
**ALAMUT**	N/A	N/A	N/A	Prevents donor site (2/5)	N/A	N/A	N/A
**GnomAD**	N/A	N/A	0.000036	0.00007262	0.00001193	N/A	0.00001336
**ACMG Evidence of pathogenicity**	PM2	PM2, PP3	PM2, PP4	PM2, PP4	PM2, PP4	PM2, PP3, PP4	PM2
**ACMG Evidence of benign impact**	BP4	None	BP4	BP4	BP4	None	BS2, BP4
**CLASSIFICATION**	Variant of uncertain significance	Variant of uncertain significance	Variant of uncertain significance	Variant of uncertain significance	Variant of uncertain significance	Variant of uncertain significance	Variant of uncertain significance

Missense and splicing site predictions were generated using the Alamut Visual v.2.10 (https://www.interactive-biosoftware.com/alamut-visual/). This program uses the Sorting Intolerant from Tolerant (SIFT, https://sift.bii.a-star.edu.sg/), Polymorphism Phenotyping v2 (PolyPhen-2, http://genetics.bwh.harvard.edu/pph2/), and mutation Taster 2 (http://www.mutationtaster.org/) algorithms to make pathogenicity predictions of missense variants (default parameters and program options were used). Alamut also uses 5 distinct algorithms (SpliceSiteFinder-like, MaxEntScan, NNPLICE, GeneSplicer, and Human Splicing Finder) to evaluate effects of variants on the splicing process. GnomAD database was used to obtain population frequencies. "()" next to SIFT and PolyPhen-2 columns indicate Pathogenicity Scores; "()" next to the Mutation Taster column indicate p-Values; "()" next to Alamut column indicates the number of splicing site algorithms used by Alamut that reached the same prediction. N/A: Not available. ***In silico*** **tools ranges and scores**: SIFT ranges: The amino acid substitution is predicted damaging if the score is <= 0.05, and tolerated if the score is > 0.05. **Polyphen-2**: 0.0 to 0.15: Variant is predicted to be benign; 0.15 to 1.0: Variants are possibly damaging; 0.85 to 1.0: Variant is predicted to be damaging. **Mutation Taster 2**: This tool provides a probability value of the prediction; a value close to 1 indicates a high "security" of the prediction; scores below 0.5 indicate a low confidence prediction. **ACMG criteria for classifying pathogenic variants:** Moderate (PM): 2. Absent from controls (or at extremely low frequency if recessive) in Exome Sequencing Project, 1000 Genomes Project, or Exome Aggregation Consortium.

Supporting (PP): 3. Multiple lines of computational evidence support a deleterious effect on the gene or gene product (conservation, evolutionary, splicing impact, etc.). 4. Patient's phenotype or family history is highly specific for a disease with a single genetic etiology. **ACMG criteria for classifying benign variants** Strong: (BS): 2. Observed in a healthy adult individual for a recessive (homozygous), dominant (heterozygous), or X-linked (hemizygous) disorder, with full penetrance expected at an early age. Supporting (BP): 4. Multiple lines of computational evidence suggest no impact on gene or gene product (conservation, evolutionary, splicing impact, etc.)

Supp. Table S6.- **Genotypic results in 353 subjects (index cases and first and second degree relatives) analyzed in our cohort.**

		-/-	-/WT	WT/WT	-	WT	Total
**Recessive cases**	Index Cases	73	--	--	--	--	73
	Family Members	53	120	21	--	--	194
**Dominant cases**	Index Cases	--	18	--	--	--	18
	Family Members	--	39	11	--	--	50
**X-Linked Cases**	Index Cases	--	1	--	3	--	4
	Family Members	--	4	1	6	3	14
						**Total**	**353**

(-/-) : biallelic pathogenic variants; (-/WT): Heterozygous pathogenic variant; (WT/WT): Homozygous wild type; (-):Hemizygous pathogenic variant; (WT): Hemizygous wild type.

TABLE 2 References

1. Simonelli, F., Testa, F., de Crecchio, G., Rinaldi, E., Hutchinson, A., Atkinson, A., …Allikmets R. (2000). New ABCR mutations and clinical phenotype in Italian patients with Stargardt disease. *Investigative Ophthalmology & Visual Science*, 41, 892-897.

2. Collin, R. W., Safieh, C., Littink, K. W., Shalev, S. A., Garzozi, H. J., Rizel, L., …Ben-Yosef, T. (2010). Mutations in C2ORF71 cause autosomal-recessive retinitis pigmentosa. *American Journal of Human Genetics*, 86, 783-788.

3. Tuson, M., Marfany, G., & Gonzàlez-Duarte R. (2004). Mutation of CERKL, a novel human ceramide kinase gene, causes autosomal recessive retinitis pigmentosa (RP26). *American Journal of Human Genetics*, 74, 128-38.

4. den Hollander, A. I., ten Brink, J. B., de Kok, Y. J, van Soest, S,, van den Born, L. I., van Driel, M. A., … Bergen, A. A. (1999). Mutations in a human homologue of Drosophila crumbs cause retinitis pigmentosa (RP12). *Nature Genetics*, 23, 217-221.

5. Zenteno. J. C., Buentello-Volante, B., Ayala-Ramirez, R., & Villanueva-Mendoza, C. (2011). Homozygosity mapping identifies the Crumbs homologue 1 (Crb1) gene as responsible for a recessive syndrome of retinitis pigmentosa and nanophthalmos. *American Journal of Medical Genetics A*, 155A, 1001-1006.

6. Xu, M., Yang, L., Wang, F., Li, H., Wang, X., Wang, W., … Chen, R. (2015). Mutations in human IFT140 cause non-syndromic retinal degeneration. *Human Genetics*, 134, 1069-1078.

7. Dryja, T. P., Rucinski, D. E., Chen, S. H., & Berson, E. L. (1999). Frequency of mutations in the gene encoding the alpha subunit of rod cGMP-phosphodiesterase in autosomal recessive retinitis pigmentosa. *Investigative Ophthalmology & Visual Science*, 40, 1859-1865.

8. Sothilingam, V., Garcia Garrido, M., Jiao, K., Buena-Atienza, E., Sahaboglu, A., Trifunović, D., … Paquet-Durand, F. (2015). Retinitis pigmentosa: impact of different Pde6a point mutations on the disease phenotype. *Human Molecular Genetics*, 24, 5486-5499.

9. Gonzalez-Fernandez, F., Kurz, D., Bao, Y., Newman, S., Conway, B. P., Young, J. E., …Khani, S. C. (1999). 11-cis retinol dehydrogenase mutations as a major cause of the congenital night-blindness disorder known as fundus albipunctatus. *Molecular Vision*, 5, 41.

10. Chacon-Camacho, O. F., Jitskii, S., Buentello-Volante, B., Quevedo-Martinez, J., & Zenteno, J. C. (2013). Exome sequencing identifies RDH12 compound heterozygous mutations in a family with severe retinitis pigmentosa. *Gene*, 528, 178-182.

11. Perrault, I., Hanein, S., Gerber, S., Barbet, F. , Ducroq, D., Dollfus, H., …Rozet, J. M. (2004). Retinal dehydrogenase 12 (RDH12) mutations in Leber congenital amaurosis. *American Journal of Human Genetics*, 75, 639-646.

12. Wang, H., den Hollander, A. I., Moayedi, Y., Abulimiti, A., Li, Y., Collin, R. W., …Chen R. (2009). Mutations in SPATA7 cause Leber congenital amaurosis and juvenile retinitis pigmentosa. *American Journal of Human Genetics*, 84, 380-387.

13. Aller, E., Nájera, C., Millán, J. M., Oltra, J. S., Pérez-Garrigues, H., Vilela, C., … Beneyto, M. (2004). Genetic analysis of 2299delG and C759F mutations (USH2A) in patients with visual and/or auditory impairments. *European Journal of Human Genetics*, 12, 407-410.

14. Aller, E., Jaijo, T., Beneyto, M., Nájera, C., Oltra, S., Ayuso, C., …Millán JM. (2006). Identification of 14 novel mutations in the long isoform of USH2A in Spanish patients with Usher syndrome type II. *Journal of Medical Genetics*, 43, e55.

15. McGee, T. L., Seyedahmadi, B. J., Sweeney, M. O., Dryja, T. P., & Berson, E. L. (2010). Novel mutations in the long isoform of the USH2A gene in patients with Usher syndrome type II or non-syndromic retinitis pigmentosa. *Journal of Medical Genetics*, 47, 499-506.

16. Huang, L., Mao, Y., Yang, J., Li, Y., Li, Y., & Yang, Z. (2018) Mutation screening of the USH2A gene in retinitis pigmentosa and USHER patients in a Han Chinese population. *Eye* (Lond) 32, 1608-1614.

17. Coppieters, F., Leroy, B. P., Beysen, D., Hellemans, J., De Bosscher, K., Haegeman, G., … De Baere E. (2007). Recurrent mutation in the first zinc finger of the orphan nuclear receptor NR2E3 causes autosomal dominant retinitis pigmentosa. *American Journal of Human Genetics*, 81, 147-157.

18. DeAngelis, M. M., Grimsby, J. L., Sandberg, M. A., Berson, E. L., & Dryja TP. (2002). Novel mutations in the NRL gene and and associated clinical findings in patients with dominant retinitis pigmentosa. *Archives of Ophthalmology*, 120, 369-75.

19. Martínez-Gimeno, M., Gamundi, M. J., Hernan, I., Maseras, M., Millá, E., Ayuso, C., … Carballo M. (2003). Mutations in the pre-mRNA splicing-factor genes PRPF3, PRPF8, and PRPF31 in Spanish families with autosomal dominant retinitis pigmentosa. *Investigative Ophthalmology & Visual Science*, 44, 2171-2177.

20. Vincent, A.L., Abeysekera, N., van Bysterveldt, K. A., Oliver, V. F., Ellingford, J. M., Barton, S., & Black, G. C. (2017). Next-generation sequencing targeted disease panel in rod-cone retinal dystrophies in Māori and Polynesian reveals novel changes and a common founder mutation. *Clinical & Experimental Ophthalmology*, 45, 901-910.

21. Villanueva, A., Willer, J. R., Bryois, J., Dermitzakis, E. T., Katsanis, N., & Davis, E. E. (2014). Whole exome sequencing of a dominant retinitis pigmentosa family identifies a novel deletion in PRPF31. *Investigative Ophthalmology & Visual Science*, 55, 2121-2129.

22. Vaithinathan, R, Berson, E. L., & Dryja, T. P. (1994). Further screening of the rhodopsin gene in patients with autosomal dominant retinitis pigmentosa. *Genomics*, 21, 461-463.

23. Matias-Florentino, M., Ayala-Ramirez, R., Graue-Wiechers, F., & Zenteno, J. C. (2009) Molecular screening of rhodopsin and peripherin/RDS genes in Mexican families with autosomal dominant retinitis pigmentosa. *Current Eye Research*, 34, 1050-1056

24. Pierce, E. A., Quinn, T., Meehan, T., McGee, T. L., Berson, E. L., & Dryja, T. P. (1999). Mutations in a gene encoding a new oxygen-regulated photoreceptor protein cause dominant retinitis pigmentosa. *Nature Genetics*, 22, 248-254.

25. Xu, Y., Guan, L., Shen, T., Zhang, J., Xiao, X., Jiang, H., … Zhang, Q. (2014). Mutations of 60 known causative genes in 157 families with retinitis pigmentosa based on exome sequencing. *Human Genetics*, 133, 1255–1271.

26. Allikmets, R., Shroyer, N. F., Singh, N., Seddon, J. M., Lewis, R. A., Bernstein, P. S., …Leppert, M. (1997). Mutation of the Stargardt disease gene (ABCR) in age-related macular degeneration. *Science*, 277, 1805-1807.

27.- Hariri, A. H., Gui, W., O'Keefe, G. A. D., Ip, M. S., Sadda, S. R., & Gorin, M. B. (2017). Ultra-widefield fundus autofluorescence imaging of patients with retinitis pigmentosa. A standardized grading system in different genotypes. *Ophthalmology Retina*, 2018, 2, 735-745.

28. Xu, M., Yang, L., Wang, F., Li, H., Wang, X., Wang, W., … Chen R. (2015). Mutations in human IFT140 cause non-syndromic retinal degeneration. *Human Genetics*, 134, 1069–1078.

29. Mackay, D. S., Dev Borman, A., Moradi, P., Henderson, R. H., Li, Z., Wright, G. A., … Moore, A. T. (2011). RDH12 retinopathy: novel mutations and phenotypic description. *Molecular Vision*, 17, 2706-2716.

30. Simovich, M. J., Miller, B., Ezzeldin, H., Kirkland, B. T., McLeod G. , Fulmer, C., … Pittler, S. J. (2001). Four novel mutations in the RPE65 gene in patients with Leber congenital amaurosis. *Human Mutation*, 18, 164.

31. Hull, S., Holder, G. E., Robson, A. G., Mukherjee, R., Michaelides, M., Webster, A. R., & Moore, A. T. (2016). Preserved visual function in retinal dystrophy due to hypomorphic RPE65 mutations. *British Journal of Ophthalmology*, 100, 1499-1505.

32. Hanein, S., Perrault, I., Gerber, S., Tanguy, G., Barbet, F., Ducroq, D., … Kaplan J. (2004). Leber congenital amaurosis: comprehensive survey of the genetic heterogeneity, refinement of the clinical definition, and genotype-phenotype correlations as a strategy for molecular diagnosis. *Human Mutation*, 23, 306-317.

33. Abu-Safieh. L., Alrashed, M., Anazi, S., Alkuraya, H., Khan, A. O., Al-Owain, M., …Alkuraya, F. S. (2013). Autozygome-guided exome sequencing in retinal dystrophy patients reveals pathogenetic mutations and novel candidate disease genes. *Genome Research*, 23, 236-247.

34. Ávila-Fernández, A., Cantalapiedra, D., Aller, E., Vallespín, E., Aguirre-Lambán, J., Blanco-Kelly, F., ...Ayuso, C. (2010). Mutation analysis of 272 Spanish families affected by autosomal recessive retinitis pigmentosa using a genotyping microarray. *Molecular Vision*,16, 2550-2558.

35. Zhao, C., Bellur, D. L., Lu, S., Zhao, F., Grassi, M. A., Bowne, S. J., … Larsson, C. (2009). Autosomal-dominant retinitis pigmentosa caused by a mutation in SNRNP200, a gene required for unwinding of U4/U6 snRNAs. *American Journal of Human Genetics*, 85, 617-627.

36. Stone, E. M. (2007). Leber congenital amaurosis - a model for efficient genetic testing of heterogeneous disorders: LXIV Edward Jackson Memorial Lecture. *American Journal of Ophthalmology*, 144, 791-811.

37. Lotery, A. J., Jacobson, S. G., Fishman, G. A., Weleber, R. G., Fulton, A. B., Namperumalsamy, P., … Stone, E. M. (2001). Mutations in the CRB1 gene cause Leber congenital amaurosis. *Archives of Ophthalmology*, 119, 415-420.

38. Corton, M., Tatu, S. D., Avila-Fernandez, A., Vallespín, E., Tapias, I., Cantalapiedra, D., …Ayuso, C. (2013). High frequency of CRB1 mutations as cause of Early-Onset Retinal Dystrophies in the Spanish population. *Orphanet Journal of Rare Diseases*, 8, 20.

39. de Castro-Miró, M., Tonda, R., Escudero-Ferruz, P., Andrés, R., Mayor-Lorenzo, A., Castro, J., … Gonzàlez-Duarte, R. (2016). Novel Candidate Genes and a Wide Spectrum of Structural and Point Mutations Responsible for Inherited Retinal Dystrophies Revealed by Exome Sequencing. *PLoS One*, 11, e0168966.

40. Eisenberger, T., Neuhaus, C., Khan, A. O., Decker, C., Preising, M. N, Friedburg, C., … Bolz HJ. (2013). Increasing the yield in targeted next-generation sequencing by implicating CNV analysis, non-coding exons and the overall variant load: the example of retinal dystrophies. *PLoS One*, 8, e78496.

41. Morimura, H., Fishman, G. A., Grover, S. A., Fulton, A. B., Berson, E. L., & Dryja, T. P. (1998). Mutations in the RPE65 gene in patients with autosomal recessive retinitis pigmentosa or leber congenital amaurosis. *Proceedings of the National Academy of Sciences U S A*, 95, 3088-3093.

42. Gu, S. M., Thompson, D. A., Srikumari, C. R., Lorenz, B., Finckh, U., Nicoletti, A. …Gal, A. (1997). Mutations in RPE65 cause autosomal recessive childhood-onset severe retinal dystrophy. *Nature Genetics*, 17, 194-197.

43. Alapati, A., Goetz, K., Suk, J., Navani, M., Al-Tarouti, A., Jayasundera, T., … Ayyagari, R. (2014). Molecular diagnostic testing by eyeGENE: analysis of patients with hereditary retinal dystrophy phenotypes involving central vision loss. *Investigative Ophthalmology and Visual Science*, 55, 5510-5521.

44. Henderson, R. H., Mackay, D. S, Li, Z., Moradi, P., Sergouniotis, P., Russell-Eggitt, I., … Moore, A. T. (2011). Phenotypic variability in patients with retinal dystrophies due to mutations in CRB1. *British Journal of Ophthalmology*, 95, 811-817.

45. Bujakowska, K., Audo, I., Mohand-Saïd, S., Lancelot, M. E., Antonio, A., Germain, A. …Zeitz, C. (2012). CRB1 mutations in inherited retinal dystrophies. *Human Mutation*, 33, 306-315.

46. Allikmets, R., Singh, N., Sun, H., Shroyer, N. F., Hutchinson, A., Chidambaram, A., … Lupski, J. R. (1997). A photoreceptor cell-specific ATP-binding transporter gene (ABCR) is mutated in recessive Stargardt macular dystrophy. *Nature Genetics*, 15, 236-246.

47. Paloma, E., Martínez-Mir, A., Vilageliu, L., Gonzàlez-Duarte, R., & Balcells, S. (2001). Spectrum of ABCA4 (ABCR) gene mutations in Spanish patients with autosomal recessive macular dystrophies. *Human Mutation*, 17, 504-510.

48. Chacón-Camacho, O. F., Granillo-Alvarez, M., Ayala-Ramírez, R., & Zenteno, J. C. (2013). ABCA4 mutational spectrum in Mexican patients with Stargardt disease: Identification of 12 novel mutations and evidence of a founder effect for the common p.A1773V mutation. *Experimental Eye Research*, 109, 77-82.

49. Cohn, A. C., Turnbull, C., Ruddle, J. B., Guymer, R. H., Kearns, L. S., Staffieri, S., …Mackey, D. A. (2011). Best's macular dystrophy in Australia: phenotypic profile and identification of novel BEST1 mutations. *Eye*, 25, 208-217.

50. Yang, Z., Chen, Y., Lillo, C., Chien, J., Yu, Z., Michaelides, M., … Zhang, K. (2008). Mutant prominin 1 found in patients with macular degeneration disrupts photoreceptor disk morphogenesis in mice. *Journal of Clinical Investigation*, 118, 2908-2916.

51. Wang, X., Feng, Y., Li, J., Zhang, W., Wang, J., Lewis, R. A., & Wong, L. J. (2016). Retinal Diseases Caused by Mutations in Genes Not Specifically Associated with the Clinical Diagnosis. *PLoS One*,11, e0165405.

52. Rivera-Brugués, N., Albrecht, B., Wieczorek, D., Schmidt, H., Keller, T., Göhring. I., …Hempel M. (2011). Cohen syndrome diagnosis using whole genome arrays. *Journal of Medical Genetics*, 48, 136-140.

53. Kolehmainen, J., Wilkinson, R., Lehesjoki, A. E., Chandler, K., Kivitie-Kallio, S., Clayton-Smith, J., … Manson, F. D. (2004). Delineation of Cohen syndrome following a large-scale genotype-phenotype screen. *American Journal of Human Genetics*, 75, 122-127.

54. Adato, A., Vreugde, S., Joensuu, T., Avidan, N., Hamalainen, R., Belenkiy, O., … Lancet, D. (2002). USH3A transcripts encode clarin-1, a four-transmembrane-domain protein with a possible role in sensory synapses. *European Journal of Human Genetics*, 10, 339-350.

55. Bernal, S., Ayuso, C., Antiñolo, G., Gimenez, A., Borrego, S., Trujillo, M. J., … Baiget, M. (2003). Mutations in USH2A in Spanish patients with autosomal recessive retinitis pigmentosa: high prevalence and phenotypic variation. *Journal of Medical Genetics*, 40, e8.
